# TALENs-directed knockout of the full-length transcription factor Nrf1α that represses malignant behaviour of human hepatocellular carcinoma (HepG2) cells

**DOI:** 10.1038/srep23775

**Published:** 2016-04-11

**Authors:** Yonggang Ren, Lu Qiu, Fenglin Lü, Xufang Ru, Shaojun Li, Yuancai Xiang, Siwang Yu, Yiguo Zhang

**Affiliations:** 1 The Laboratory of Cell Biochemistry and Topogenetic Regulation, College of Bioengineering and Faculty of Sciences, Chongqing University, No. 174 Shazheng Street, Shapingba District, Chongqing 400044, China; 2 State Key Laboratory of Natural and Biomimetic Drugs, and Department of Chemical Biology, School of Pharmaceutical Sciences, Peking University Health Science Center, No 38 Xueyuan Rd., Haidian District, Beijing 100191, China

## Abstract

The full-length Nrf1α is processed into distinct isoforms, which together regulate genes essential for maintaining cellular homeostasis and organ integrity, and liver-specific loss of Nrf1 in mice results in spontaneous hepatoma. Herein, we report that the human constitutive Nrf1α, rather than smaller Nrf1β/γ, expression is attenuated or abolished in the case of low-differentiated high-metastatic hepatocellular carcinomas. Therefore, Nrf1α is of importance in the physio-pathological origin and development, but its specific pathobiological function(s) remains elusive. To address this, TALENs-directed knockout of Nrf1α, but not Nrf1β/γ, is created in the human hepatocellular carcinoma (HepG2) cells. The resulting *Nrf1α*^−*/*−^ cells are elongated, with slender spindle-shapes and enlarged gaps between cells observed under scanning electron microscope. When compared with wild-type controls, the invasive and migratory abilities of *Nrf1α*^−*/*−^ cells are increased significantly, along with the cell-cycle G2-M arrest and S-phase reduction, as accompanied by suppressed apoptosis. Despite a modest increase in the soft-agar colony formation of *Nrf1α*^−*/*−^ cells, its loss-of-function markedly promotes malgrowth of the subcutaneous carcinoma xenograft in nude mice with hepatic metastasis. Together with molecular expression results, we thus suppose requirement of Nrf1α (and major derivates) for gene regulatory mechanisms repressing cancer cell process (e.g. EMT) and malignant behaviour (e.g. migration).

To maintain cellular homeostasis and physiological integrity of life systems, all organisms living in oxygenated environments have evolutionally developed efficient cytoprotective strategies against a vast variety of stresses (e.g. oxidants, xenobiotics, nutrients), pathophysilogical stimuli (e.g. inflammation and aging) and other biological cues (e.g. metabolites, inducers and inhibitors)^1^,^2^,^3^,^4^. Of note, antioxidant, detoxification and cytoprotective responses signaling towards cognate gene regulatory networks, such as those controlling metazoan development and organ homeostasis, are monitored principally by the cap’n’collar (CNC) basic-region leucine zipper (bZIP) family of transcription factors^5^,^6^,^7^,^8^. This family comprises the founding *Drosophila* Cnc protein, the *Caenorhabditis elegans* Skn-1 protein, the vertebrate activator nuclear factor-erythroid 2 (NF-E2) p45 and its related factors Nrf1 [including transcription factor 11 (TCF11, which is a longer isoform of Nrf1), and Locus control region-factor 1 (LCR-F1, a short isoform also called Nrf1β)], Nrf2 and Nrf3, as well as the transcription repressors Bach1 and Bach2. In all cases except Skn-1, CNC-bZIP proteins heterodimerize with small Maf or other bZIP proteins before they bind to antioxidant and/or electrophile response element (ARE/EpRE) sequences in their target gene promoters. As a result, this family of transcription factors control critical homeostatic and developmental pathways because they regulate both basal and inducible expression of ARE/EpRE-battery genes, which encode antioxidant proteins, detoxification enzymes, metabolic enzymes and 26S proteosomal subunits^9^,^10^,^11^.

Amongst the mammalian Nrf factors, NF-E2 p45 and Nrf3 are subject to tissue-specific expression in haematopoietic and placental cell lineages, respectively^12^,^13^,^14^. By contrast, Nrf1 and Nrf2 are ubiquitously expressed and thus represent two principal CNC-bZIP factors that regulate ARE-driven cytoprotective genes in various tissues^15^,^16^,^17^. Of note, Nrf2 is well-documented as a master regulator of adaptive responses to oxidative stressors and electrophiles^16^,^18^. However, Nrf2 is not essential for normal growth and development. This is supported by the fact that global knockout of its gene in mice yields viable animals^19^, and whilst *Nrf2*^−/−^ mice do not spontaneously develop cancer, they are more susceptible than wild-type mice to chemical carcinogens^20^. Although induction of Nrf2 has been considered as a chemopreventive target^16^,^21^,^22^, which is supported by the finding that its cytoprotective effect against carcinogenesis is enhanced by forced expression of a constitutively dominant-active caNrf2 factor in transgenic mice^23^. However, it is of significant importance to note that the basal expression of ARE-driven genes, but not their inducible expression, is crucial for anti-tumour chemoprevention against DMBA + TPA-induced carcinogenesis in additional transgenic mice that express a dominant-negative dnNrf2 factor, which may also inhibit other Nrf/CNC factors (e.g. Nrf1)^24^. Conversely, permanently hyperactive Nrf2 is also thought of as an unrecognized mediator of oncogenesis and promotes cancer cell survival and tumourigenesis^25^,^26^,^27^. Therefore, the dual-opposing roles of Nrf2 in tumor prevention and progression have led us to take account of its bidirectional potentials to implicate in cancer treatment, as described elsewhere^28^,^29^.

By contrast with Nrf2, relatively less is known about Nrf1 (refs 5,6,15). Such being the case, Nrf1 possesses a remarkable feature that gains a sharp distinction from Nrf2, which is defined by the fact that Nrf2 is dispensable for development due to no obvious phenotype exhibited in its knockout mice^19^, whilst Nrf1 is essential for maintaining cellular homeostasis and organ integrity during development and growth because distinct gene-targeting strategies for knockout of *Nrf1* (also called *nfe2l1*) in mice are enabled to yield various animal lines with several significant pathological phenotypes^30^,^31^,^32^,^33^,^34^,^35^. Global knockout of *Nrf1* (by distinct gene-targeting strategies) in the mouse leads to variable lethality of unviable embryos between 6.5 and 14.5 days post-coitus, resulting from severe oxidative stress^30^,^31^,^32^. The phenotypic examination demonstrates that loss of Nrf1’s function cannot be compensated by the presence of Nrf2, albeit both CNC-bZIP factor possesses certain overlapping functions in regulating ARE-driven gene expression as confirmed by double knockout *(Nrf1*^−*/*−^*:Nrf2*^−*/*−^) animal model^36^. Further, tissue-specifically conditional knockout of *Nrf1* (by the Cre-loxP system) in the mouse liver, pancreas, brain and bone results in distinct pathologies of non-alcoholic steatohepatitis (NASH) and hepatoma^33^,^34^, Type-2 diabetes^37^, neurodegeneration^38^,^39^ and reduced bone size^40^, respectively. These pathological phenotypes are also accompanied by significant disorders of glucose, lipid and protein metabolisms. The notion is supported by further experiments revealing that inducible knockout of *Nrf1* in the mouse liver^35^ and its gain-of-function (by over-expressing *Nrf1-Tg*) in the transgenic mice^41^ cause impaired expression of key genes responsible for glucose and lipid metabolisms, leading to the pathogenesis of NASH and diabetes mellitus, respectively. Collectively, these findings demonstrate that Nrf1 (and/or its isoforms) fulfils a unique and indispensable biological function(s) that is distinctive from that of Nrf2, in maintaining cellular metabolic homeostasis and normal organ integrity. However, it is unknown which isoforms (e.g. Nrf1α, Nrf1β/LCR-F1, Nrf1γ and Nrf1δ, with their diagrams illustrated in Fig. 1) contribute to its pathobiological function(s), in particular cytoprotection against carcinogenesis, because none of the single isoform-specific knockout models are available.

The sharp functional distinction between Nrf1 and Nrf2 is largely determined by differences in their molecular and cellular basis. By contrast with the single soluble Nrf2 protein, Nrf1 is identified as a membrane-bound CNC-bZIP factor with dynamic topologies integrated within the proximity of the endoplasmic reticulum (ER) and nuclear envelope membranes, and is also processed to yield multiple isoforms that dictate its overall activity to tempo-spatially fine-tune transcriptional expression of cognate target genes^15^,^42^,^43^,^44^. Accumulating evidence reveals that at least eleven Nrf1 isoforms are produced from the single *nfe2l1* gene, though differentially expressed, in differential mammalian species^5^,^45^,^46^,^47^,^48^,^49^,^50^,^51^,^52^. These isoforms are synthesized by translation through distinct initiation signals (i.e. the first or internal start ATG codons) embedded in different lengths of open reading frames, some portions of which can be alternatively spliced from the cognate mRNAs^45^,^46^,^47^,^49^,^50^,^53^. The prototypic full-length Nrf1α protein arises by alternative splicing of the mRNA enabling translation of the long TCF11 formy^47^,^48^, such that Nrf1α lacks the Neh4L subdomain (aa 242–271, see Fig. 1c) of TCF11, which is rarely expressed in the human cancer cells (unpublished data) and also is not expressed in the mouse^30^,^31^,^45^,^46^,^54^. Despite removal of the Neh4L subdomain from the putative tansactivation domain (TAD) in Nrf1α, this factor was shown to have a similar ability to transactivate ARE-driven genes as TCF11 (with a molecular mass of approximately 140-kDa estimated on Laemmli SDS-PAGE gels)^55^. Both the full-length Nrf1α and longer TCF11 proteins can also be subject to the ER-associated topogenesis and selective post-translational processing to yield distinct isoforms of between 120-kDa and 25-kDa (which are estimated on LDS-NuPAGE gels)^44^,^56^,^57^. Amongst these isoforms, the mouse 120-kDa Nrf1α glycoprotein is thought to be inactive because its TAD elements are buried in the ER lumen, whilst dynamic repositioning of the TADs into the cyto/nucleoplasm enables Nrf1α to be deglycosylated insomuch as to function as an active 95-kDa factor (despite a possible mixture with a fraction of 95-kDa non-glycosylated proteins). Furthermore, other isoforms of between about 85-kDa and 55-kDa are postulated to be active processed forms because they lack the ER-anchoring N-terminal domain (NTD, aa 1-124, that negatively regulates Nrf1), but retain essential portions of TADs or *en bloc*^44^,^56^,^57^. These proteins may also be further processed to give rise to various TAD-deficient isoforms, such as those of approximately 55-kDa, 36-kDa and 25-kDa (designated Nrf1β/LCR-F1, Nrf1γ and Nrf1δ, respectively)^5^,^15^,^42^,^43^,^58^. Albeit these N-terminally truncated variants are neither targeted to the ER nor recovered in membrane fractions^58^,^59^, Nrf1β/LCR-F1 only functions a weak activator because it lacks its acidic domain 1 (AD1, which is a major TAD element), whilst Nrf1γ and Nrf1δ act as dominant-negative inhibitors competing against wild-type Nrf1 (and/or Nrf2)^43^,^44^,^46^,^49^,^50^. Collectively, these short isoforms are generated primarily by the prototypic Nrf1α processing at both post-transcriptional and post-translational levels, expect that an additional fraction of such short proteins (e.g. Nrf1β/LCR-F1 and Nrf1γ) are produced though internal translation pathway on the base of the fact that the putative products are significantly diminished by mutation of relevant in-frame translation start codons^42^,^44^,^55^. However, the individual isoform-specific function(s) in pathophysiology remains to be elucidated.

Since the aforementioned facts demonstrate that the full-length Nrf1α is selectively processed into distinct isoforms, which together finely-tune expression of genes essential for sustaining cellular homeostasis and physiological integrity, it is inferable to be of crucial importance in the origin and development. The functional loss of Nrf1 (including Nrf1α and Nrf1β/γ) in the mouse liver leads to spontaneous development of hepatoma^33^,^34^. Notably, no polypeptides specifically corresponding to the Nrf1α and its longer products (topoforms) are detected in the human erythroleukemia (K562) cells, with an exception of Nrf1β expressed as a major endogenous protein^45^. Thereby, Nrf1α rather than Nrf1β is postulated to confer a *bona fide* cytoprotective effect on hosts against carcinogenesis and malignant transformation. To address this hypothesis, this study attempts to determine whether Nrf1α plays a specific role in the cytoprotection from malignant deterioration of human cancer cells. First of all, transcription activator-like effector nucleases (TALENs)-directed frameshift mutation into the genomic *Nrf1* sequence is allowed for site-specific deletion of Nrf1α, but not of other smaller isoforms including Nrf1β/γ, in the human hepatocellular carcinoma (HepG2) cells, followed by selection of the homozygous knockout (*Nrf1α*^−*/*−^) monoclonal cells. Subsequently, we have herein examined whether: (i) the resulting *Nrf1α*^−*/*−^ cell morphology, cell-cycle phases and apoptosis are changed when compared with the wild-type controls; (ii) both the invasive and migratory abilities of cancer cells are affected by Nrf1α-specific knockout; (iii) Nrf1α-specific knockout causes an alteration to the soft agar colony formation of *Nrf1α*^−*/*−^ cells and the *in vivo* growth of subcutaneous carcinoma xenograft derived from *Nrf1α*^−*/*−^ cells in nude mice; (iv) loss of its putative function leads to dysregulated expression of a subset of key genes controlling cell process and behaviour (e.g. proliferation, migration, invasion and cytoskeleton) in both *Nrf1α*^−*/*−^ cells and derived xenograft mice; and (v) the basal constitutive expression of Nrf1*α* (and its derivates) is suppressed to low levels in the human hepatocellular carcinoma tissues and relevant cancer cell lines. Consequently, our evidence that has been provided herein reveals that Nrf1α is endowed with the potential as a chemopreventive target against malignant development of liver cancer.

## Results

### Construction of Nrf1α-specific frameshift mutants by TALENs-mediated genome editing of the target sequence in model HEK293 cells

**Figure 2:**
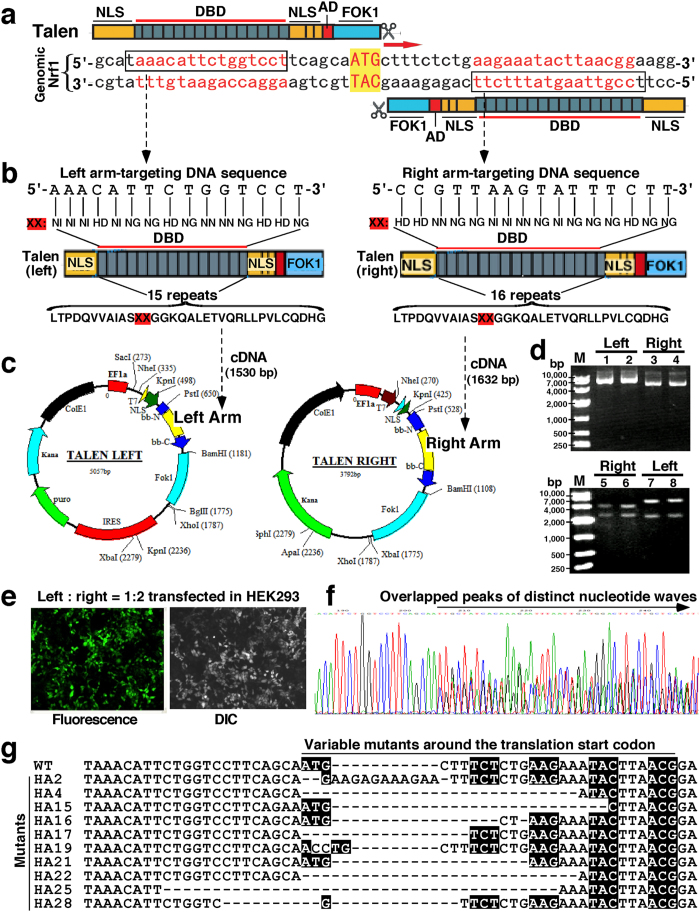
Construction of TALENs-expressing plasmids applied in the human *Nrf1* gene editing. (**a**) Schematic diagram of TALENs-mediated editing of the human genomic *Nrf1* sequence. A pair of TALEN-left and TALEN-right [either comprises nuclear localization signal (NLS), DNA-binding domain (DBD), activation domain (AD) and the fusion nuclease FOK1] were designed to recognize the *boxed* target sequences in close proximity to the site responsible for the translation start codon. (**b**) An assembly of the repeat modular DBD-coding cDNA sequences was made according to the ‘protein-DNA’ code as a guiding principle^64^,^65^,^66^. Each repeat module amino acid sequence (*lower row*) of the DBD encompasses the indicated hypervarible diresidues (**XX**) in positions 12 and 13 that have a capability to bind a specific nucleotide within the target sequence (i.e. the ‘protein-DNA’ code illustrated in *the upper two rows*). (**c**) The assembled cDNA sequences for DBDs of TALEN-left and TALEN-right were inserted into indicated sites, respectively. (**d**) The expression constructs for TALEN-left (6587 bp) and TALEN-right (5425 bp) (*upper panel*), together with their PstI/BamHI-digested fragments (*lower panel*), were identified by their electrophoretic mobility on 0.8% agar gel, before being sequenced to ensure the fidelity of the inserted DBDs-coding cDNA fragments. (**e**) HEK293 cells were co-transfected for 6 h with TALEN-left and TALEN-right constructs (at a ratio of 1:2), along with a GFP expression plasmid. The cells were then selected by treatment with puromycin (2 μg/ml) for 48 h before being subjected to the cloning of single cells grown in 96-well plates, in order to determine the activity of TALENs-mediated gene editing. (**f**) The genomic DNA from the individual cell clones served as a template of PCR to amplify the TALENs-target region of *Nrf1*, followed by sequencing of PCR products. The result revealed that overlapped peaks of distinct nucleotide waves started around the specific site responsible for translation of *Nrf1α*. (**g**) A nucleotide alignment of the human wild-type (WT) *Nrf1* and its frameshift mutants around and within TALENs-target sequences. The deletion nucleotides were indicated by dashed dots. The putative cDNA codons were placed in the black backgrounds.

The TALENs-targeted genome editing is widely applied for site-specific alterations of nearly all genes of interest in a broad range of cell types and organisms^60^,^61^,^62^. The high-efficiency of TALENs is dictated by targetable nucleases composed of a customizable sequence-specific DNA-binding domain (DBD) fused C-terminally to an effector nuclease domain of Fok1 (Fig. 2a). The latter Fok1 acts as a functional dimer required for its nuclease activity to cleave DNA in a non-sequence-specific manner such that double-strand breaks are induced within specific DNA sites, and ensuing DNA-repair mechanisms (i.e. non-homologous end-joining) can be exploited to create genetic alterations (e.g. deletion, insertion or others) of targeted genomic sequence at the putative cleavage site^60^,^63^,^64^. Therefore, TALENs-mediated genome editing of the human *Nrf1* sequence was here employed to introduce Nrf1α-reading frameshift mutation into the gene locus, which was allowed for desirable interruption of the open reading frame after and around the translation start ATG codon within its full-length transcript (Fig. 2a, *indicated by arrows*).

In attempt to induce double-strand breaks with a desire to create Nrf1α-reading frameshift mutation, a pair of TALENs-based constructs (called left-arm and right-arm, which recognize 5′-TAAACATTCTGGTCCT-3′ and 5′-TCCGTTAAGTATTTCTT-3′, respectively) were made to meet the requirement for homodimerization of their Fok1 nuclease domains being positioned to adjacent genomic target sites in close proximity to the translation start codon within an 18-bp spacer 5′-TCAGCAATGCTTTCTCTG-3′, situated between the above two TALENs-targeting DNA sites (Fig. 2a,b). The editing of one-to-one correspondences between each of the hypervariable diresidues (i.e. NN, NI, HD and NG) in the repeat modular DBD of TALENs and the indicated individual bases [i.e. guanine (G), adenine (A), cytosine (C) and thymine (T), expect for the first conserved T that has been positioned just 5′ to the nucleotide fragment] in the target Nrf1 sequence was designed according to a guiding principle (Fig. 2b), called the ‘protein-DNA’ code^64^,^65^,^66^. Subsequently, the designed cDNA products encoding the repeat modular DBDs were cloned to create two expression constructs for TALEN-Left and TALEN-Right (Fig. 2c) before being subjected to the sequencing in order to confirm the fidelity of their DBD fragments inserted (Fig. 2d).

To evaluate the activity of TALENs to introduce the putative mutation into the genomic *Nrf1* sequence, the model HEK293 cells were co-transfected for 6 h with the above pair of TALEN-Left and TALEN-Right constructs (at a ratio of 1:2, total cDNA of 4.5 μg) and were then transferred in the fresh selection medium containing 2 μg/ml of puromycin (which enables almost all untransfected control cells to be killed within 48 h) (Fig. 2e). The puromycin-resistant cells were selected before being subjected to the genomic DNA extraction and subsequent amplification by polymerase chain reaction (PCR, with a pair of primers: 5′-CGAGAAGGGAAAGTGAATG-3′ and 5′-CTGGGTCTGAGTATAGGCA-3′), followed by cloning of PCR products in the pMD19-T plasmid. As anticipated, the sequencing of PCR products showed that overlapped peaks of distinct nucleotide waves start to emerge from around the translation start codon (Fig. 2f). Further sequencing of relevant single-cell clones revealed that the TALENs-directed mutagenesis system is available insofar as to yield Nrf1α-reading frameshift mutations (i.e. deletion and insertion) occurring at the designed sites (Fig. 2g).

### The homozygous bi-allelic knockout (Nrf1α^−/−^) from the HepG2-based monoclonal cell lines established by using TALENs-directed mutagenesis system

**Figure 3:**
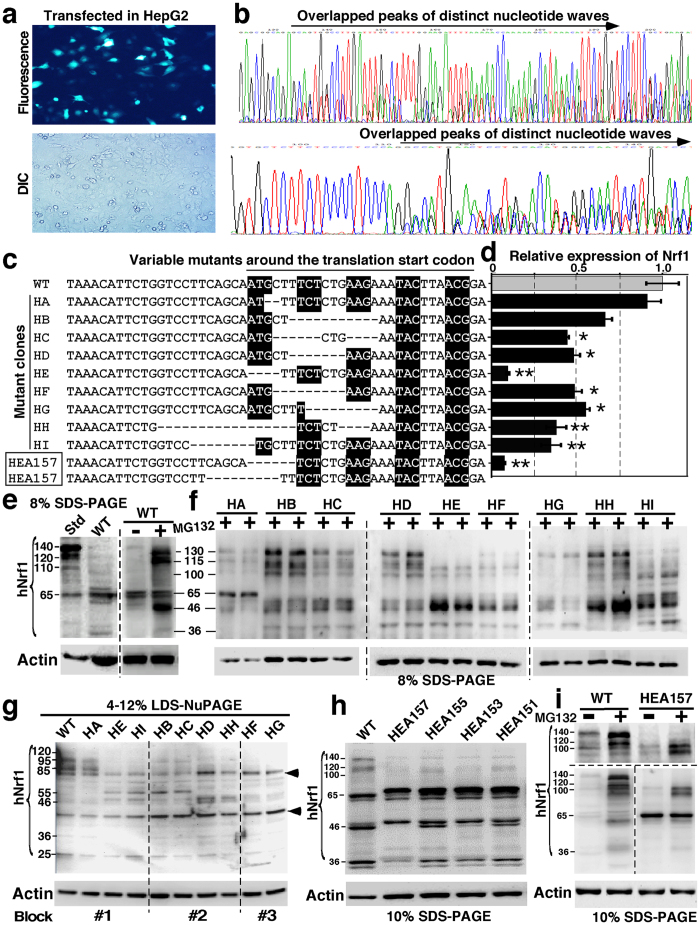
Establishment of the homozygous *Nrf1α*^−*/*−^ knockout monoclonal cell line by using TALENs. (**a**) HepG2 cells were co-transfected with TALEN-left (1.5 μg) and TALEN-right (3 μg) constructs (along with 0.5 μg of a GFP-expressing plasmid to verify the transfection efficacy). The cells were selected by 2.5 μg/ml puromycin for 48 h before being subjected to the single cell cloning in 96-well plates, in order to establish the homozygous *Nrf1α*^−*/*−^ knockout monoclonal cell line. (**b**) The genomic DNA from the individual cell clones served as a template of PCR to amplify TALENs-recognized region of *Nrf1*. The sequencing result revealed overlapped peaks of distinct nucleotide waves starting around the site responsible for the initial translation of *Nrf1α*. (**c**) A nucleotide alignment of human wild-type (WT) *Nrf1* and its frameshift mutants around TALENs-target sequences. The deletion nucleotides were represented by dashed dots. The putative cDNA codons were placed in the black backgrounds. (**d**) Different mRNA levels of *Nrf1* in TALENs-mediated mutant cell lines (called HA to HI) and wild-type HepG2 cells were measured by quantitative real-time PCR. The results were calculated as a fold change (mean ± S.E.) of Nrf1 transcriptional expression. Significant decreases (*p < 0.05, **p < 0.01, n = 9) are indicated, relative to the wild-type control value of 1 measured from HepG2 cells. (**e**,**f**) Total lysates of each cell lines that had been treated with MG132 or untreated were subjected to protein separation by 8% Laemmli SDS-PAGE gels running in the pH 8.9 Tris-glycine buffer, followed by immunoblotting with Nrf1 antibodies to determine the protein expression patterns. The standard (Std) sample was made from human Nrf1-overexpressing cells. (**g**) The lysates (re-grouped into 3 blocks) were subjected to further protein resolution by 4–12% LDS-NuPAGE gels running in the pH 7.3 MES buffer. (**h**,**i**) The homozygous *Nrf1α*^−*/*−^-specific knockout monoclonal cell lines (e.g. HEA157) were established on the base of the heterozygous mutant HA cells. The cell lines were further identified by sequencing of TALENs-target genomic DNA (**c**), quantitative real-time PCR (**d**), and western blotting with different two antibodies against Nrf1 that had been isolated by 10% SDS-PAGE gels (**h**,**i**).

To bypass a predictable obstacle that Nrf1α-reading frameshift mutations are allowed to abolish the expression of TCF11 in addition to Nrf1α *per se*, thus it is better for us to have chosen the human hepatocellular carcinoma (HepG2) cells that express a major full-length protein of Nrf1α rather than TCF11 (which is also lost in the mouse^30^,^31^,^45^,^46^,^54^), in order to establish an Nrf1α-specific kockout cell line by using the TALEN system. For this reason, HepG2 cells were co-transfected for 6 h with the pair of TALEN-Left and TALEN-Right expression constructs as described above and then selected with puromycin (2.5 μg/ml) for 48 h (Fig. 3a). The puromycin-resistant cells were subjected to monoclonal selection of single cells that were subcultured in each of the 96-well plates. The monoclonal cells were identified by sequencing of the genomic *Nrf1* nucleotides, in order to confirm the frameshift mutations occurring within the TALENs-targeted region, that was amplified by PCR (with a pair of primers: 5′-CGAGAAGGGAAAGTGAATG-3′ and 5′-CTGGGTCTGAGTATAGGCA-3′) (Fig. 3b).

Following monoclonal selection, sixteen of mutant cell lines were chosen for the continuous subculture to give rise to mono-allelic (*Nrf1α*^+*/*−^) or bi-allelic (*Nrf1α*^−*/*−^) knockout monoclonal cell lines, nine of which (called HA to HI) were identified by sequencing of the genomic *Nrf1* mutants at the putative TALENs-targeted sites (Fig. 3c). The expression of these mutants at both mRNA and protein levels was further determined by real-time qPCR (with a pair of primers 5′-GCTGGACACCATCCTGAATC-3′ and 5′-CCTTCTGCTTCATCTGTCGC -3′) (Fig. 3d) and western blotting of Nrf1α protein derivates (Fig. 3e–g), respectively. Amongst these mutants, an optimal monoclonal cell line (HE) bearing only 4-bp deletion was based for re-transfection with the expression constructs for TALEN-Left and TALEN-Right and further selection by puromycin as described above, in order to establish as a homozygous Nrf1α-specific knockout cell model. Of note, one of four bi-allelic knockout monoclonal cell lines was designated as HEA157 (*Nrf1α*^−*/*−^), with a 6-bp deletion positioned in an allele and additional 9-bp deletion positioned in another allele (Fig. 3c). However, it is intriguing that such short deletions around the first translation start codon of *Nrf1α* in the genomic loci have led to a significant decrease in the entire basal mRNA expression of Nrf1 (including almost all isoforms) to ~10% of control values measured from wild-type cells (Fig. 3d), although the detailed mechanism is unknown.

When compared with ectopic wild-type proteins (including Nrf1α derivates between 140-kDa and 100-kDa, and smaller isoforms such as 65-kDa Nrf1β and 36-kDa Nrf1γ), relatively lower levels of the equivalent endogenous proteins except Nrf1β/γ were expressed in HepG2 cells (Fig. 3e, *cf. Std with WT lanes in left panel*). By contrast, significant increases in the abundance of endogenous Nrf1 proteins at estimated masses of 130-kDa, 115-kDa and 46-kDa were observed following treatment of cells with the proteasomal inhibitor MG132 (5 μmol/L) (Fig. 3e, *right panel*). This observation, together with our previous work^43^,^44^, suggests that endogenous 130-kDa Nrf1α (and 65-kDa Nrf1β) is an unstable protein such that it is degraded possibly through the proteasome-mediated pathway to give rise to several smaller isoforms of between 130-kDa and 46-kDa.

To examine which monoclonal cell lines (HA to HI) are mono-allelic or bi-allelic Nrf1α-specific knockout mutants, total lysates of each cell lines that had been treated with MG132 or untreated were subjected to protein separation by Laemmli SDS-PAGE gels containing 8% polyacrylamide in the pH 8.9 Tris-glycine running buffer (Fig. 3f) or by LDS-NuPAGE gels containing 4–12% polyacrylamide in the pH 7.3 MES running buffer (Fig. 3g). Although similar proteins exhibited distinct electrophoretic mobility as reported previously^44^,^67^, western blotting results together with the genomic nucleotide sequencing revealed that knockout mutants in HB, HC, HD and HH cell lines are mono-allelic because a small fraction of Nrf1α-related proteins were retained, whilst other mutants in HA, HF, HG and HI cell lines are predicted to be heterozygous bi-allelic, but only HE mutant is homozygous bi-allelic with a very low level of Nrf1 mRNA being expressed (Fig. 3d). Subsequently, four of HE-derived monoclonal cell lines (including HEA157) were further optimized and identified by immunoblotting with two different antibodies against Nrf1 (Fig. 3h,i). The results unraveled that none of the longer Nrf1α-related proteins of between 140-kDa and 110-kDa, rather smaller isoforms Nrf1β/γ, were expressed in HEA157 cells, although two additional smaller mutant proteins (estimated close to ~100-kDa, which is predicted to be a product from translation of Nrf1^ΔN^ transcript and thus allowed for immunoreaction with anti-Nrf1 antibody) have emerged instead (Fig. 3i). Together with the sequencing results, HEA157 is considered as a homozygous bi-allelic Nrf1α-specific knockout cell line, and hence is used in the following experiments in order to determine whether Nrf1α plays a role in cytoprotecting against malignant transformation of cancer.

**Figure 4:**
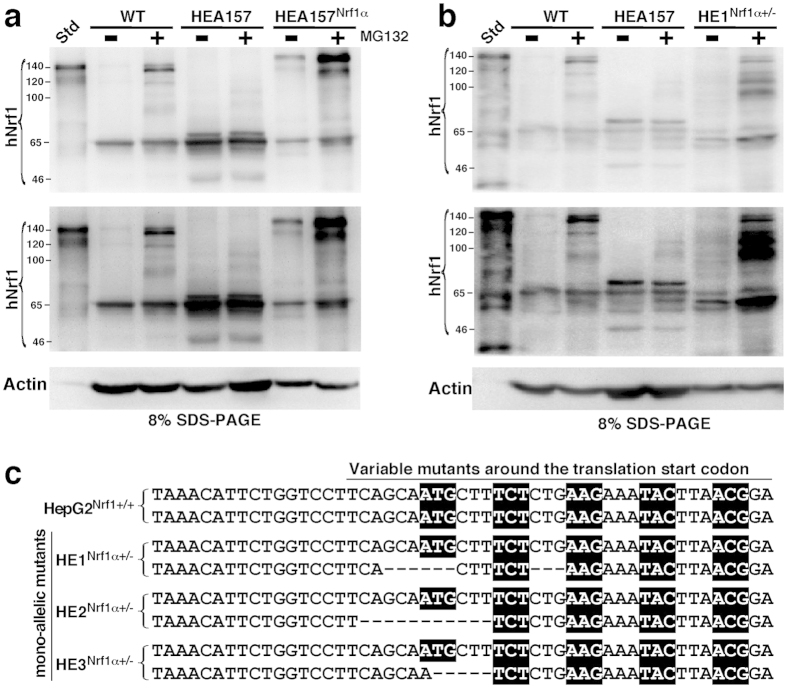
Stable restoration of intact Nrf1α proteins into its knockout cells that are bi- or mono-allelic mutants. (**a**) A lentivirally-packaged Nrf1α-expressing construct (Lenti-pEZ-Lv203) was transfected, according to the manufacturers’ instructions, into the bi-allelic knockout (*Nrf1α*^−*/*−^) monoclonal cell line (HEA157), and thus the Nrf1α-restored cell line was designated as HEA157^Nrf1α^. Subsequently, western blotting of HEA157^Nrf1α^ and its parent cell lines that had been treated with or without MG132, revealed that stably forced expression of Nrf1α and its derivate proteins was accompanied by a relative decrease of Nrf1β when compared with their expression levels measured in both HEA157 and HepG2 cell lines. The upper two panels show the images obtained from different exposure to X-ray. Of note, other detailed descriptions of HEA157^Nrf1α^ had been not focused herein. (**b**,**c**) Three mono-allelic knockout (*Nrf1α*^+*/*−^) monoclonal cell lines (called HE1^Nrf1α+/−^, HE2^Nrf1α+/−^ and HE3^Nrf1α+/−^) had been identified by western blotting (**b**, and see Fig. S1), target DNA sequencing (**c**), and other cell biology data (see the legends of Figs S2 to S4). These data are a representative of at least three independent experiments undertaken on separate occasions that were each performed in triplicate. (**c**) A nucleotide alignment of human wild-type (WT) *Nrf1* and its mono-allelic mutants around the translation start codons that are targeted by TALENs and confirmed by DNA sequencing.

The validity of the above TALEN constructs targeting for genomic DNA site-specific deletion of the constitutive Nrf1α expression was assessed by restoring the ectopically-expressing wild-type Nrf1α into the *Nrf1α*^−*/*−^ HEA157 cells (Fig. 4a). The resulting HEA157^Nrf1α^ cells showed a similar pattern of Nrf1 proteins to those expressed endogenously in the wild-type HepG2 or ectopically-expressing Nrf1 in distinct control cells (i.e. *WT and Std* in Fig. 4a). Notably, the validation of TALENs-directed genomic deletion of Nrf1α expression has also been supported by the data obtained from the mono-allelic knockout mutant (i.e. *Nrf1α*^+*/*−^) cell lines (designated HE1^Nrf1α+*/*−^ to HE3^Nrf1α+*/*−^, see Fig. 4b,c and supplemental Figs S1 to S4), and further works focused on the bi-allelic knockout mutant (*Nrf1α*^−*/*−^) HEA157 cells rather than on the Nrf1α-restored HEA157^Nrf1α^ cells (so that more detailed results from being rescued by HEA157^Nrf1α^ will be not shown herein).

### Loss of Nrf1α leads to obvious phenotypic changes in the morphology of hepatoma cells

**Figure 5:**
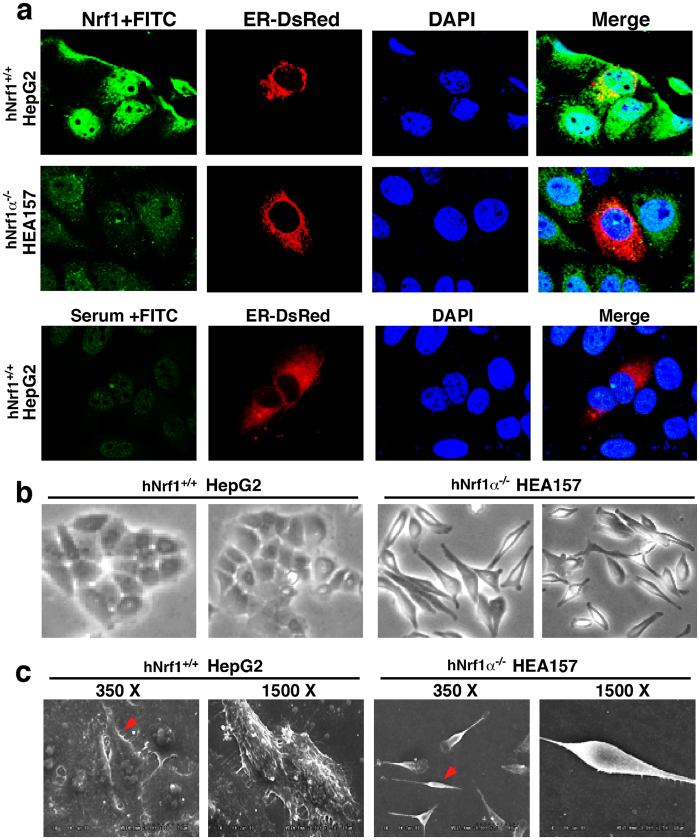
Obvious phenotypic changes in the morphology of *Nrf1*α^−/−^ hepatoma cells. (**a**) HepG2 (*Nrf1α*^+*/*+^) and HEA157 (*Nrf1α*^−*/*−^) cells were transfected with 1.5 μg of an expression construct for ER-DsRed marker protein. After the cells were allowed to recover for 12 h, subcellular location of Nrf1 was examined by immuocytochemistry with anti-Nrf1 antibody (*the upper two rows*, it should be noted that the anti-Nrf1 antibody is replaced by normal serum as an internal control in *the third row*), followed by confocal imaging. FITC-labelled second antibody was used to locate Nrf1 proteins. Nuclear DNA was stained by DAPI. The ER/DsRed gave a red image positively in the ER. The merge signal represents the results obtained when the three images were superimposed. (**b**,**c**) The above cells were subjected to observation of the morphological changes by light microscopy (**b**) and scanning electron microscopy (**c**), before relevant cell images were acquired. Overall, these images shown with different magnifications in sizes are a representative of at least three independent experiments undertaken on separate occasions that were each performed in triplicate (n = 9). The red arrowed cell was magnified to1500× than their original sizes (*cf. right image with left image*).

Confocal microscopy imaging revealed that the green immunofluorescent signals representing endogenous Nrf1 are distributed in the cytoplasm and nucleus of human HepG2 cells (Fig. 5a). Of note, the green image of Nrf1 stained in the cytoplasm of HepG2 cells is superimposed with the red fluorescent signal of the ER-DsRed marker (Fig. 5a, *upper panel row*); this is consistent with our previous results obtained from the rat liver RL-34 cells^58^, indicating that a cytoplamic portion of Nrf1α (and relevant derivate topoforms) is localized primarily in the ER. By contrast, knockout of Nrf1α caused a significant decrease in the green staining of Nrf1 in HEA157 cells (Fig. 5a, *middle panel row*). The residual signals presented in HEA157 cells may, at least in part, be attributable to the remaining expression of smaller isoforms (including 100-kDa polypeptide and Nrf1β/γ) in the Nrf1α-deficient cells (Fig. 3h,i), in addition to the non-specific staining signal similar to that obtained from the normal rabbit serum instead of anti-Nrf1 antibody in the immunocytochemistry experiments (Fig. 5a, *lower panel row*).

To investigate effects of Nrf1α-specific knockout on the morphology of human hepatoma cells, *Nrf1α*^−*/*−^ HEA157 cells together with wild-type (*Nrf1*^+/+^) HepG2 cells were subjected to visualization of cell shapes by both general light microscopy (Fig. 5b) and scanning electron microscopy (Fig. 5c). In contrast with the round-like (ellipse) shapes of wild-type HepG2 cells that are epithelial in morphology, loss of Nrf1α enables its deficient HEA157 cells to be shrunken in size, but the *Nrf1α*^−*/*−^ cell shapes are elongated with slender spindle-like forms (Fig. 5b,c). Intriguingly, some cytoplasmic projections (i.e. lamellipodia and filopodia) from the rough surface of *Nrf1*^+*/*+^ HepG2 cells and the surface constitutive domains (e.g. canalicular, sinusoidal or microvillus-like structures) were observed under scanning electron microscope (Fig. 5c, *left two panels*), whilst these epithelial surface structures disappeared from the smooth surface of *Nrf1α*^−*/*−^ HEA157 cells (Fig. 5c, *right two panels*). The latter cell-cell interaction appeared to decrease such that their junction gaps were conversely increased by the absence of Nrf1α in HEA157 cells and thus the spindle-shaped *Nrf1α*^−*/*−^ cells interacted with each other possibly through focal points. Collectively, the phenotypic differences in the morphology of between *Nrf1*^+*/*+^ and *Nrf1α*^−*/*−^ cells indicate a possibility that Nrf1α-specific knockout may promote the epithelial-mesenchymal transition (EMT), a process entailing a considerable risk of cancer transformation.

### Knockout of Nrf1α enhances the migration and invasion of hepatoma cells *in vitro*

**Figure 6:**
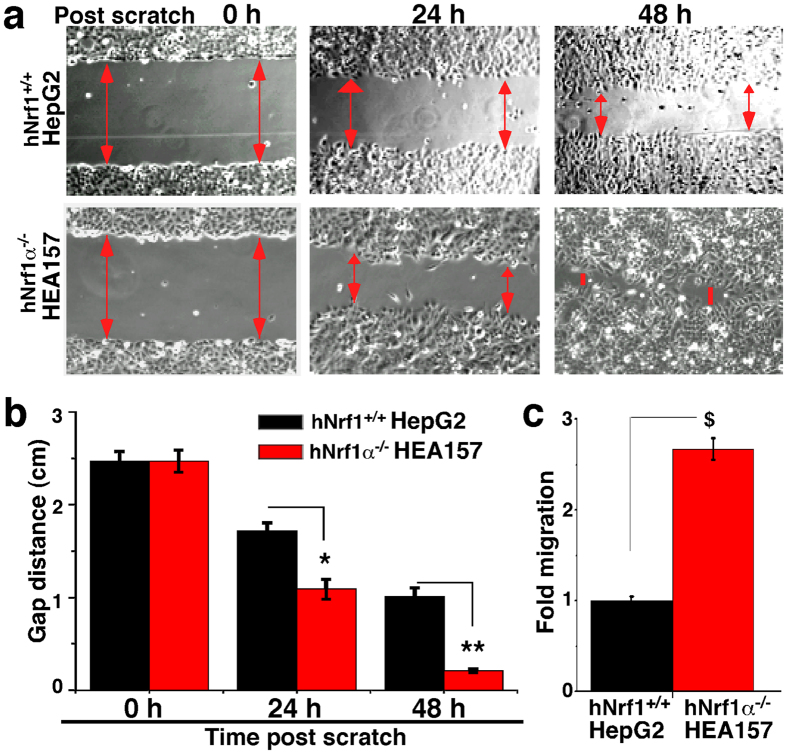
Changing migration of *Nrf1*α^−/−^ cells to close the *in vitro* scratch. HepG2 (*Nrf1α*^+*/*+^) and HEA157 (*Nrf1α*^−*/*−^) cells were starved for 12 h in a serum-free medium and then treated for additional 6 h with 1 μg/ml of mitomycin C. Subsequently, a clear ‘scratch’ was created before being allowed for being healed in the continuous culture at 37 °C with 5% CO2. The scratched images were captured at the beginning and at 12-h intervals during cell migration to close the scratch (**a**), followed by quantification of the cell migration (**b**,**c**). The results were calculated as a fold change (mean ± S.D.) of the scratched gap distance (**b**) and fold migration (**c**) of *Nrf1α*^−*/*−^cells, which are shown as a representative of at least three independent experiments undertaken on separate occasions that were each performed in triplicate. (**b**,**c**) Significant decreases (*p < 0.05, **p < 0.01, n = 9) and significant increases (^$^p < 0.05, n = 9) are indicated, relative to the corresponding control values measured from wild-type (*Nrf1α*^+*/*+^) HepG2 cells. The double arrows indicate the gap distance after the ‘scratch’ wound.

To date, it is unknown what effects Nrf1α exerts on malignant behaviour of human cancer cells. Firstly, a convenient *in vitro* scratch assay, as described by^68^, was employed to measure the extents to which the presence or absence of Nrf1α has respective effects on the migration of *Nrf1*^+*/*+^ HepG2 and *Nrf1α*^−*/*−^ HEA157 cells in the leading edges of the scratch. The scratch testing of the images revealed that loss of Nrf1α causes a quicker increase in the migration of its deficient HEA157 cells to close the scratch wound, when compared with wild-type control HepG2 cells (Fig. 6a). This is also supported by quantifying the gap distance between the leading edges of the scratch at the beginning and at intervals of 12 h during cell migration insomuch as to heal the wound (Fig. 6b, and data not shown). Then, the migration rate of *Nrf1α*^−*/*−^ HEA157 cells was calculated to be ~2.6-fold, that is significantly increased, when compared with the one-fold migration of *Nrf1*^+*/*+^ HepG2 control cells (Fig. 6c).

**Figure 7:**
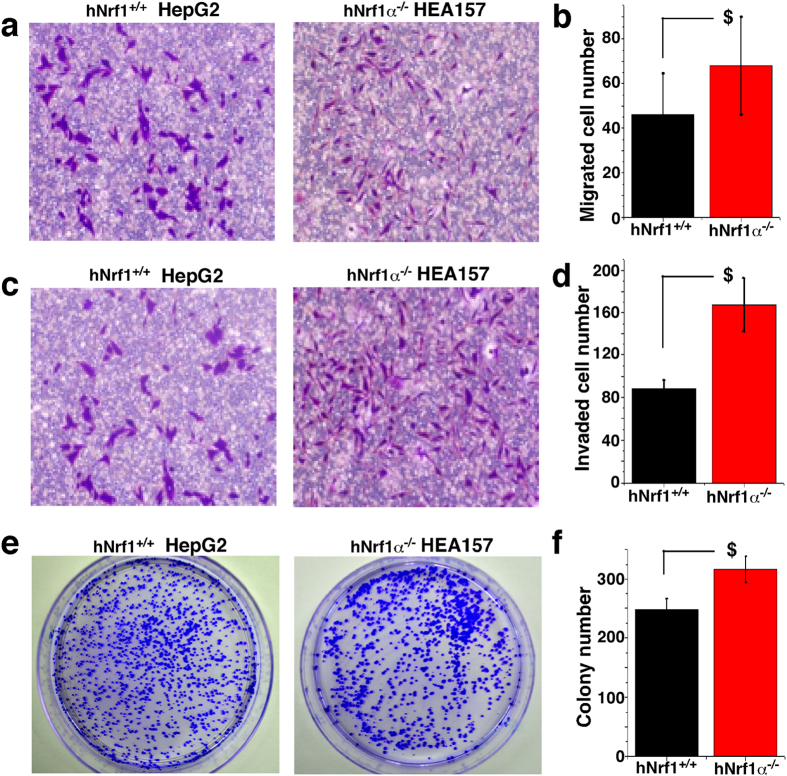
Increases in the migration and invasion of *Nrf1*α^−/−^ cells and their clone formation on soft agar. (**a–d**) HepG2 (*Nrf1α*^+*/*+^) and HEA157 (*Nrf1α*^−*/*−^) cells were starved for 12 h in a serum-free medium and then subjected to transwell migration (**a**) and invasion (**c**) assays as described in the section of ‘Materials and methods’. The migratory and/or invasive cells, that had passed through the 8-μm microporous membrane and attached to the lower surface of the transwell membranes, were fixed with 4% paraformaldehyde and stained with 1% crystal violet reagent before being counted. The results were calculated as a fold change (mean ± S.D.) of migratory (**b**) and invasive (**d**) *Nrf1α*^−*/*−^cells, which are shown as a representative of at least three independent experiments undertaken on separate occasions that were each performed in triplicate. Significant increases (^$^p < 0.05, n = 9) are indicated, relative to the corresponding control values obtained from wild-type *Nrf1α*^+*/*+^ HepG2 cells. (**e**,**f**) The soft agar colony formation of the above two cell lines was examined as described in the text of ‘Materials and Methods’. The resulting cell clones formed on the soft agar plates were stained with 1% crystal violet reagent before being counted. (**f**) The data were calculated as a fold change (mean ± S.D.) of the number of *Nrf1α*^−*/*−^ cell clone formation, and the significant increase (^$^p < 0.05, n = 9) is analyzed, relative to the control values of *Nrf1α*^+*/*+^ cells.

Secondly, *in vitro* transwell migration and invasion assays of hepatoma cells were performed as described^69^, in order to measure abilities of *Nrf1α*^−*/*−^ or *Nrf1*^+*/*+^ cells to move through the cell-permeable membrane. As shown in Fig. 7(a,c), loss of Nrf1α causes significant increases in the migratory and invasive abilities of the *Nrf1α*^−*/*−^ HEA157 cells by ~1.6-fold (Fig. 7b) and ~1.9-fold (Fig. 7d), when compared with those of *Nrf1*^+*/*+^ HepG2 control cells. Taken together, the results demonstrate that migration and invasion of hepatoma cells are enhanced by Nrf1α-specific knockout. In turn, this fact could be placed as a solid basis for us to postulate that Nrf1α is likely to repress transformation, migration and invasion of cancer cells.

### Knockout of Nrf1α promotes the transformation of its deficient cancer cells and the malignant growth of subcutaneous carcinoma xenograft in nude mice with the liver metastasis

Since anchorage-independent growth is a hallmark of carcinogenesis^70^,^71^, whether the presence or absence of Nrf1α has an effect on the ability of relevant hepatoma cells to grow independently of a solid surface was here examined by using the soft agar colony formation assay, in order to determine malignant transformation capability of *Nrf1α*^−*/*−^ HEA157 cells. By comparison with the presence of Nrf1 in HepG2 control cells (Fig. 7e), knockout of Nrf1α caused a modest increase in the number of cell colonies formed by *Nrf1α*^−*/*−^ HEA157 cells in the soft agar (Fig. 7f), most of which had each grown to such a considerable size that the bulk of the cell colonies were bunched together, as illustrated for the images (Fig. 7e, *cf. right with left panels*).

**Figure 8:**
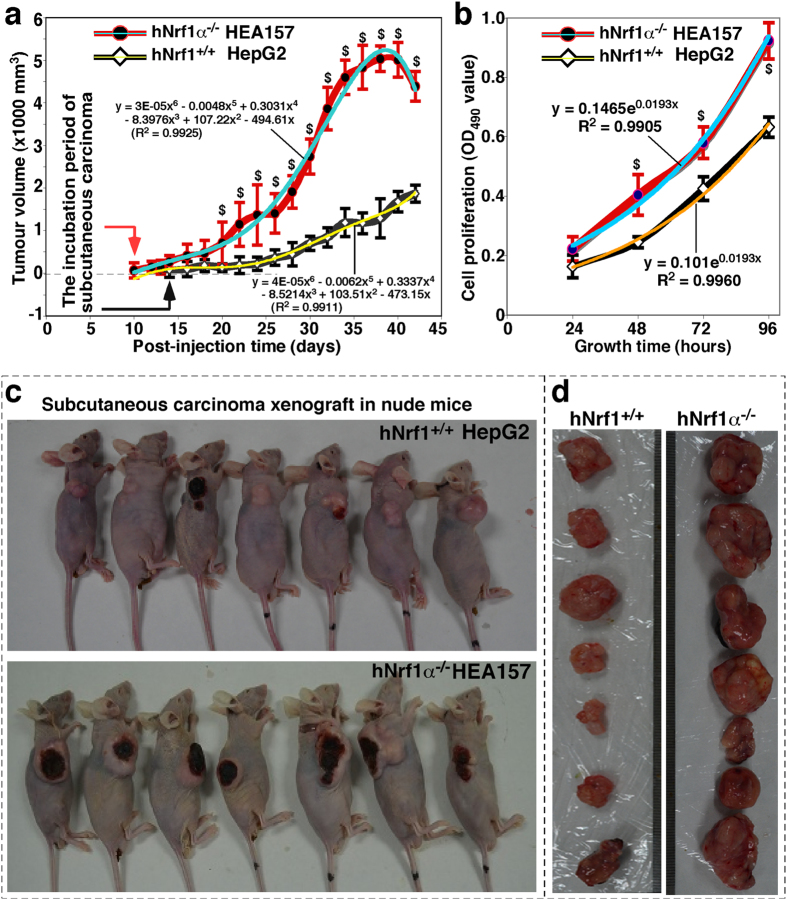
*In vivo* malgrowth of *Nrf1*α^−/−^ cells-derived subcutaneous tumour xenografts in nude mice. Either HepG2 (*Nrf1α*^+*/*+^) or HEA157 (*Nrf1α*^−*/*−^) cells that had been growing in the exponential phase were inoculated subcutaneously into male nude mice, followed by observation of the subcutaneous tumour xenografts that had emerged and developed. (**a**) Shows that the tumour sizes were successively measured until six weeks when the mice were sacrificed before the transplanted tumors were excised. The results of growing tumour sizes were calculated as a fold change (mean ± S.D.) and then are shown graphically (n = 7 per group). (**b**) Shows that the *in vitro* cell proliferation determined by MTS assay (n = 9). Significant increase (^$^p < 0.05) in the proliferation of *Nrf1α*^−*/*−^ cells and relevant exnograft tumourogenesis are indicated, relative to the control values obtained from *Nrf1α*^+*/*+^ cells. (**c**) Shows two groups of different subcutaneous tumour-bearing mice that were inoculated with either HepG2 (*Nrf1α*^+*/*+^) or HEA157 (*Nrf1α*^−*/*−^) cells. (**d**) Two groups of different xenograft tumors with different sizes were excised after the mice were sacrificed, and were also subjected to the histopathological and other examinations (shown below).

**Figure 9:**
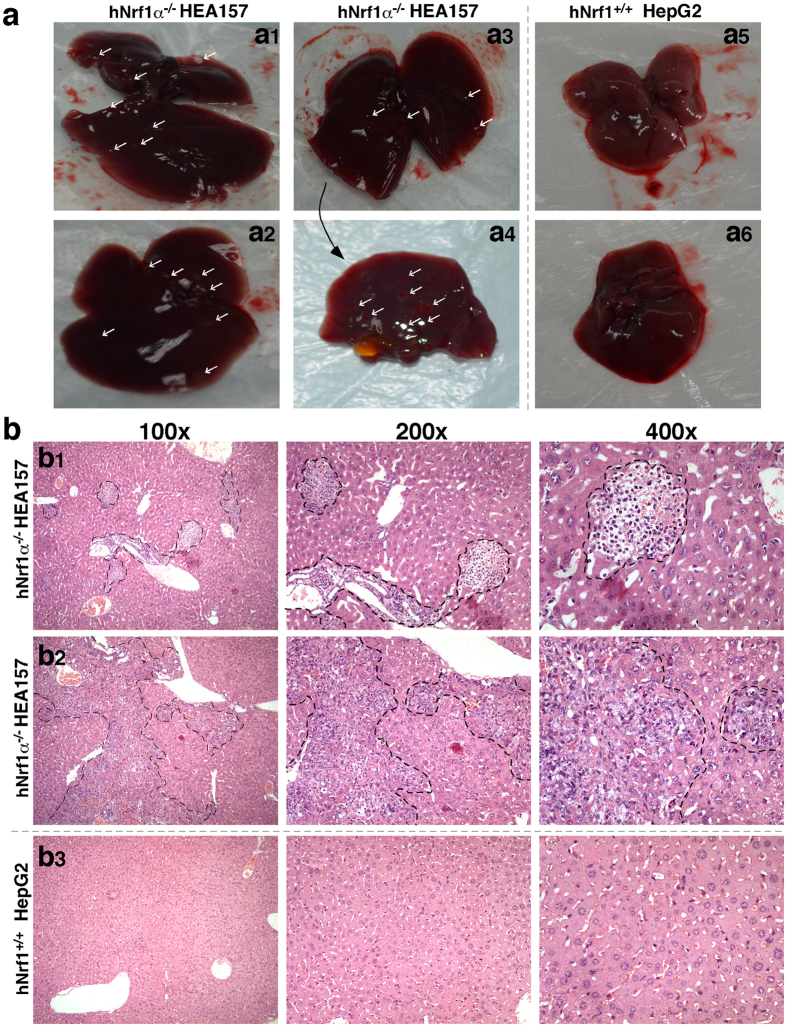
Hepatic metastasis of *Nrf1*α^−/−^ cells-derived subcutaneous tumour xenografts in nude mice. (**a**) Small metastatic tumour nodules were seen (*directed by arrows*) in the livers (but not other organs) in the subcutaneous tumour-bearing mice that had been heterotransplanted with HEA157 (*Nrf1α*^−*/*−^, a1-4), but not with HepG2 (*Nrf1α*^+*/*+^, a5-6). The anatomical section of the liver (a3) was enabled for further observation of hepatic metastasis from the inside (a4), followed by (**b**) histopathological examination by the routine hematoxylin-eosin staining (HE). The resulting images shown with different magnifications in sizes are a representative of at least three independent experiments undertaken on separate occasions that were each performed in duplicate (n = 6). The obvious areas of hepatic metastatic tumour nodules, along with cancer embolus, were roughly illustrated in the images of the livers in tumour-bearing mice that had been subcutaneously inoculated with *Nrf1α*^−*/*−^ (b1-2) rather than *Nrf1α*^+*/*+^ (b3) hepatoma cells.

Which effects Nrf1α-deficiency elicits on the carcinogenesis of human hepatocellular cancer are next determined by using an animal xenograft model, in which human hepatoma cells were heterotransplanted into immunodeficient nude mice, as described previously^72^. After either *Nrf1α*^−*/*−^ HEA157 or *Nrf1*^+*/*+^ HepG2 hepatoma cells were inoculated subcutaneously into the right upper back region of nude mice at a single site, the incubation period of carcinogenesis before the *in situ* emergence of visible tumour xenogafts derived from *Nrf1α*^−*/*−^ cells was shortened to two-thirds of that of *Nrf1*^+*/*+^ cells-derived tumour xenografts (Fig. 8a, *left panel*). These human tumour xenografts had been clearly seen until two weeks after subcutaneous inoculation of hepatoma cells into the nude mice. Within the ensuing four weeks of the cancer growth, the tumour sizes were measured at one-day intervals, and the results were calculated as shown graphically (Fig. 8a). The resultant curve displays that Nrf1α-deficient carcinoma xenografts were growing gradually within the first two weeks, but thereafter they were expanding exponentially in size until day 35, followed by a moderate growth to the maximum at day 40. Subsequently, the growing tumours ruptured insomuch as to become bleeding ulcers (Fig. 8c, *lower panel*). By contrast, the control cells-derived tumour xenografts were slowly growing in a steady rate (Fig. 8a), without bleeding ulcers being formed within six weeks of the nude mice (Fig. 8c, *upper panel*). Therefore, these results convincingly demonstrate that knockout of Nrf1α causes a significant increase in the tumour size of human carcinoma xenografts resulting from *Nrf1α*^−*/*−^ HEA157 cells, when compared with the equivalent xenografts derived from the *Nrf1*^+*/*+^ HepG2 control cells. In addition, it should also be noted that the Nrf1α-deficient carcinoma xenograft mice, rather than the control mice, suffered from a severe syndrome that resembles human cancer cachexia, as described elsewhere^73^,^74^. The occurrence of the cancer cachexia syndrome was much likely to be attributed to hepatic metastasis (Fig. 9); this pathology was accompanied by potential cancer-promoting inflammation in the livers of tumour-bearing mice injected with *Nrf1α*^−*/*−^ knockout cells. However, similar pathological changes did not appear to be examined in equivalent organs of wild-type control mice (Fig. 9).

**Figure 10:**
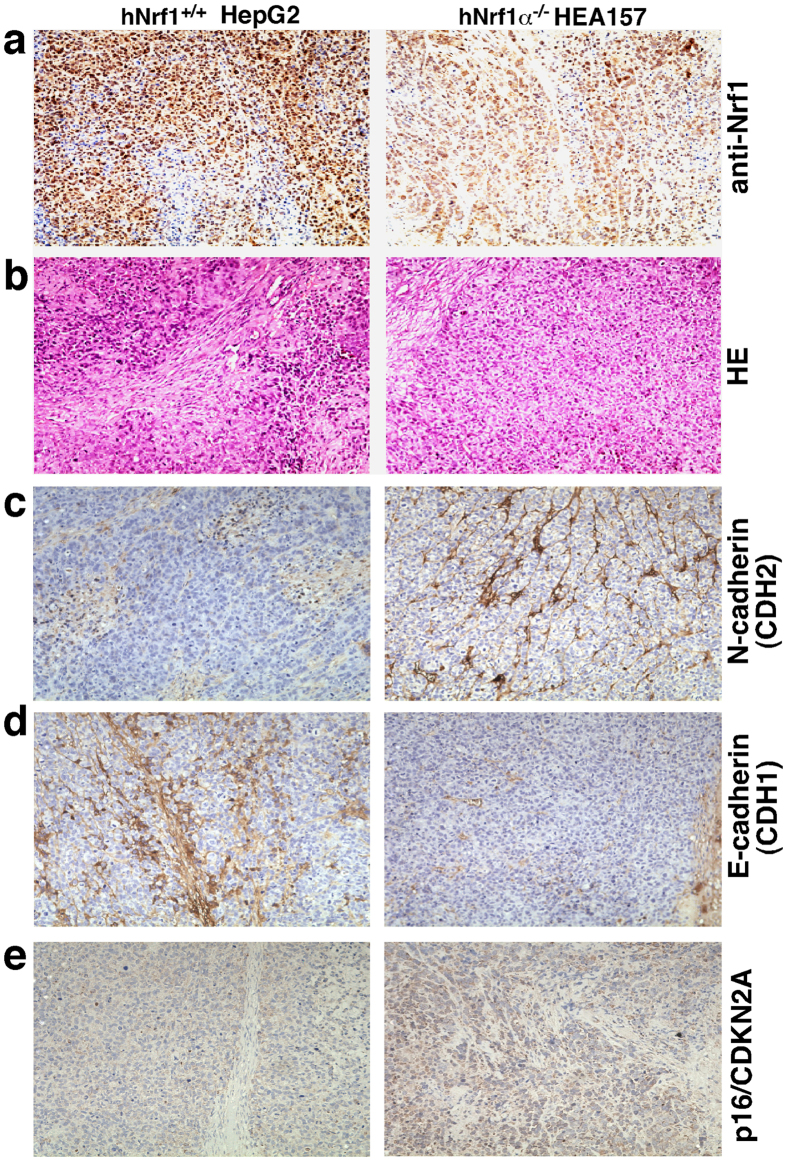
Altered expression of Nrf1 and EMT-specific markers in the *Nrf1*α^−/−^ cells-derived xenografts. After scarification of the tumour-bearing mice that had been injected with either HEA157 (*Nrf1α*^−*/*−^) or HepG2 (*Nrf1α*^+*/*+^), the subcutaneous xenograft tissues were obtained and then subjected to the routine histopathological examination by the hematoxylin-eosin staining (HE, (**b**), followed by immunohistochemical staining with antibodies against Nrf1 (**a**), the EMT- specific markers E-cadherin (CDH1, (**d**) and N-cadherin (CDH2, (**c**), as well as p16/CDKN2A (**e**). These images shown herein are a representative of at least three independent experiments undertaken on separate occasions that were each performed in duplicate (n = 6). Of note, the xenograft samples were also subjected to quantitative real-time PCR analysis of relevant gene expression (data shown in Fig. S5).

Six weeks later, the xenograft model mice were sacrificed to excise the heterotransplanted carcinoma. The resulting images illustrated that the human carcinoma derived from *Nrf1α*^−*/*−^ HEA157 cells was estimated in size to be ~3.0 times larger than that of the carcinoma derived from *Nrf1*^+*/*+^ HepG2 control cells (Fig. 8c,d). Then xenograft tumours were subjected to histopathological examination by routine hematoxylin-eosin staining, followed by immunohistochemical staining with antibodies against Nrf1 (Fig. 10a). When compared with wild-type control (*Nrf1*^+*/*+^), the expression of Nrf1 was markedly decreased in the *Nrf1α*^−*/*−^ HEA157 cells-derived tumour tissue (Fig. 10a, *right upper panel*). The remaining immunoreactive signals were contributed by cross-reacting with mouse orthologous antigen (i.e. mNrf1) that was expressed in the heterotransplanted tumour xenograft, albeit human Nrf1α was lost in Nrf1α-deficient carcinoma.

Moreover, loss of Nrf1α caused a profound increase in the *in vitro* proliferation of its knockout HEA157 cells, when compared with wild-type control (*Nrf1*^+*/*+^) cells (Fig. 8b). Subsequently, the rate of cell proliferation was calculated mathematically, demonstrating that *Nrf1α*^−*/*−^ cell growth was distinguishably from that of the control, within 96 h after experimental treatment.

Further immunohistochemistry of the EMT-specific markers E-cadherin and N-cadherin (encoded by *CDH1* and *CDH2*, respectively) revealed that a marked reduction in the expression of E-cadherin was replaced by a significant enhancement of N-cadherin expression in the *Nrf1α*^−*/*−^-derived xenograft tissues (Fig. 10c,d. *cf. right with left panels*). This observation suggests that loss of Nrf1α promotes the putative EMT process during malgrowth of cancer cells; the notion is also supported by real-time quantitative PCR data (Fig. S5). In addition, alternations in the expression levels of other signaling molecules and cell-cycle controls (Figs 10e and S5) were thoroughly described below.

### Loss of Nrf1α leads to the cell cycle alterations accompanied by suppressed apoptosis

**Figure 11:**
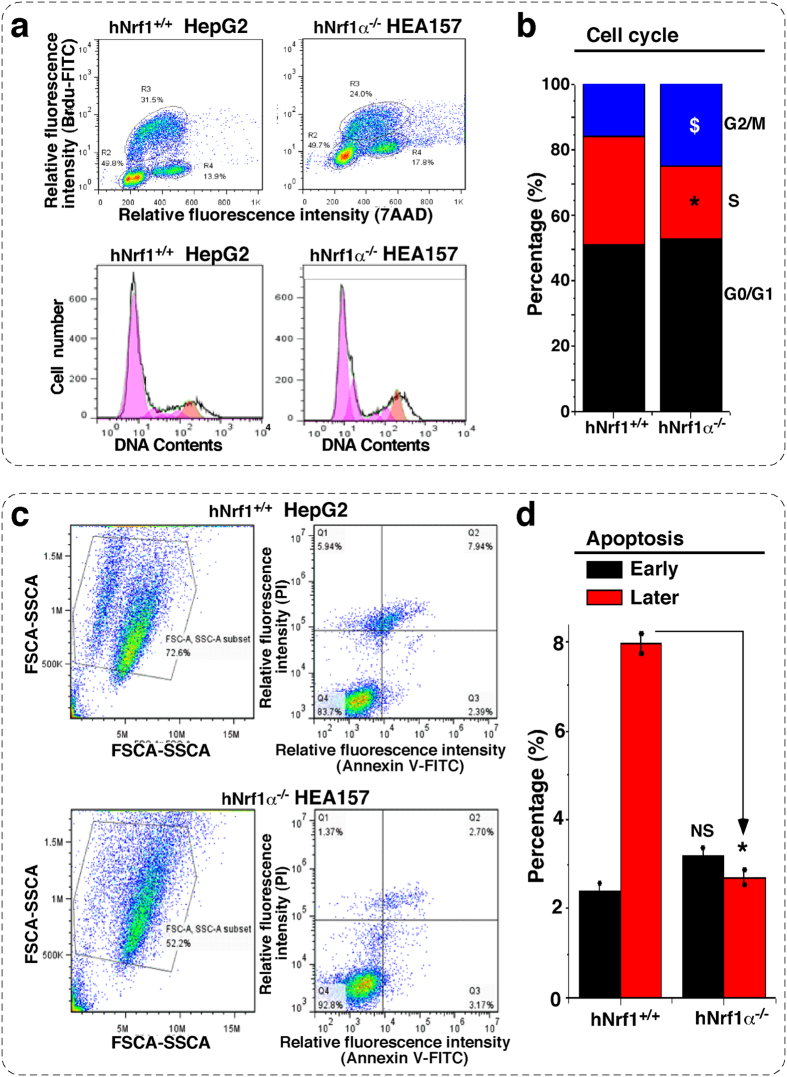
Loss of Nrf1α leads to alterations in the deficient cell cycle phases and apoptosis status. Alterations of either HepG2 (*Nrf1α*^+*/*+^) or HEA157 (*Nrf1α*^−*/*−^) cell cycle (**a**) and apoptosis (**c**) were determined by using fluorescence-activated cell sorting (FACS) with different reagents, as described in the section of ‘Materials and methods’. These cell distributions were monitored with the BD Accuri C6 software and also analyzed by the FlowJo 7.6.1 software. The results were calculated as a percentage (%, mean ± S.D.) of cells examined in different phases (**b**) or apoptosis status (**d**). Significant increase (^$^p < 0.05, n = 9) and significant decreases (*p < 0.05, n = 9) in the above alterations resulting from *Nrf1α*^−*/*−^ are indicated, relative to the corresponding control values obtained from wild-type *Nrf1α*^+*/*+^ cells. These data shown here are a representative of at least three independent experiments undertaken on separate occasions that were each performed in triplicate. NS represents no significant differences.

The above-described results demonstrate that loss of Nrf1α leads to striking enhancements in transformation, carcinogenesis and malgrowth of the carcinoma xenografts derived from *Nrf1α*^−*/*−^ HEA157 cells. Such being the case, malignant behaviour is assumed to be attributable to alterations in the cell cycle and apoptosis. To test this hypothesis, fluorescence-activated cell sorting (FACS) was employed to examine effects of *Nrf1α*^−*/*−^ on the HEA157 cell division cycle and its auto-apoptosis. The results revealed that knockout of Nrf1α caused the cell-cycle arrest at G2/M phases, inasmuch as they were increased by 6% along with an 8% reduction of the S-phase, when compared with those obtained from the control HepG2 cells (Fig. 11a,b). The cell-cycle alteration supports the notion that Nrf1α-specific knockout promotes the proliferation of its deficient HEA157 cells.

It is plausible that the S-phase reduction enables the cell division to be conversely increased, whilst the cell-cycle G2-M arrest provides the sufficient time allowed for damaged cells to be repaired before they enter mitosis or undergo apoptosis. As a consequence, the proliferation rate of *Nrf1α*^−*/*−^ HEA157 cells was elevated as described above (Figs 7e,f and 8b). Intriguingly, only a small number (8%) of auto-apoptotic cells were sorted out from hepatoma HepG2 cells by using an automatic FACS system (Fig. 11c,d). By contrast, the later apoptosis (which is associated with the cell-cycle G2-M checkpoint arrest) of HEA157 cells was modestly suppressed to ~2.2% by knockout of Nrf1α (Fig. 11c,d). This finding is consistent supportively with the notion that the cell cycle G2-M arrest facilitates the proper repair of damaged cells insomuch as to undertake normal mitosis, so that the division and proliferation of hepatoma cells are incremented by knockout of Nrf1α as accompanied by slightly reduced auto-apoptosis. However, no obvious differences in the early apoptosis and relevant cell-cycle G0/G1 phase of between *Nrf1α*^−*/*−^ and *Nrf1*^+*/*+^ cell lines were detected (Fig. 11a–d).

### Knockout of Nrf1α results in dysregulation of genes controlling the cell cycle and apoptosis

**Figure 12:**
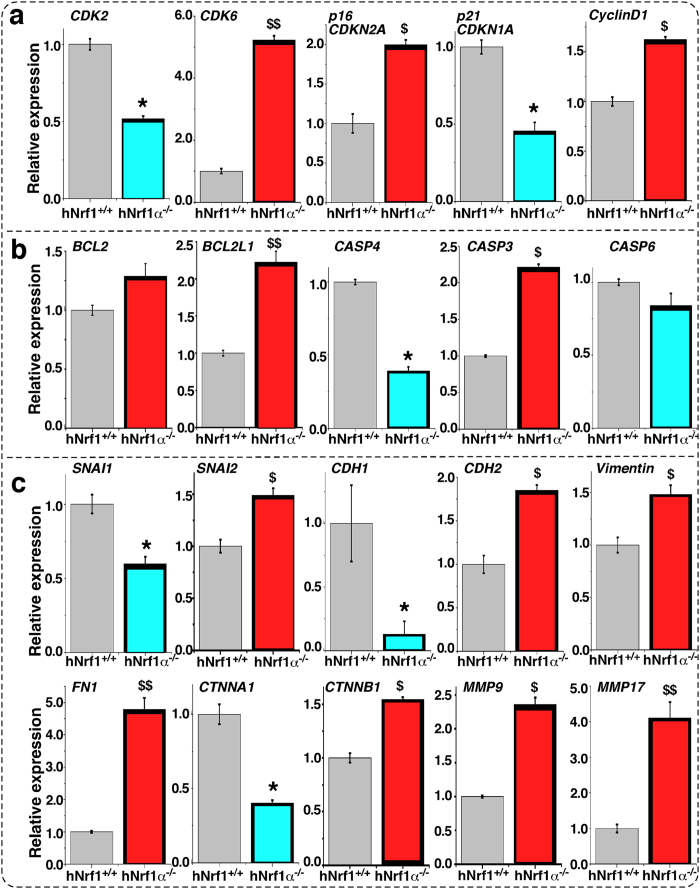
Dysregulated transcriptional expression of distinct genes in *Nrf1*α^−/−^ cells. Total RNAs were isolated from HepG2 (*Nrf1α*^+*/*+^) or HEA157 (*Nrf1α*^−*/*−^) cells and then reversely transcribed into the first strand of cDNA. Subsequently, different mRNA levels of distinct genes controlling cell process and behaviour [i.e. cell division cycle (**a**), apoptosis (**b**), migration and invasion including the EMT markers (**c**)] were measured by quantitative real-time PCR. The results were calculated as a fold change (mean ± S.E) of mRNA levels of gene expression in *Nrf1α*^−*/*−^ cells and revealed that loss of Nrf1α results in dysregulation of indicated gene transcription with significant decreases (*p < 0.05, n = 9) or significant increases (^$^p < 0.05, ^$$^p < 0.01, n = 9), relative to their basal mRNA levels of corresponding genes expressed in wild-type *Nrf1α*^+*/*+^ cells (with relevant values being defined as 1). These data shown here are a representative of at least three independent experiments undertaken on separate occasions that were each performed in triplicate.

**Figure 13:**
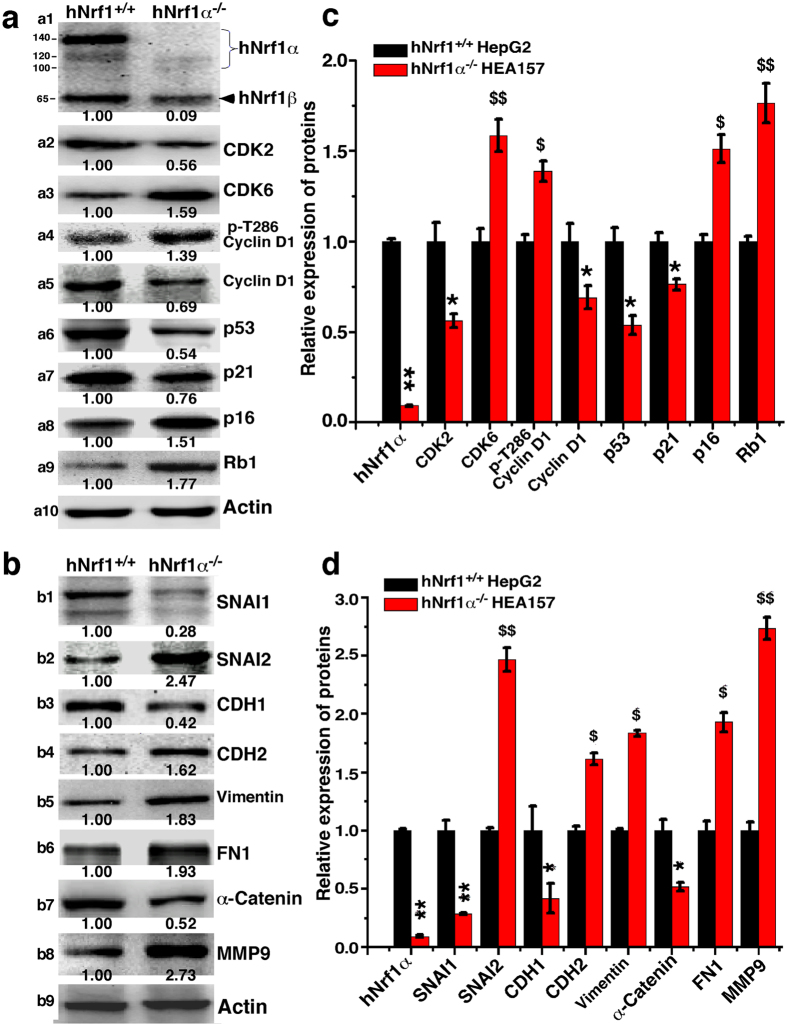
Alterations in translational expression of distinct genes in *Nrf1*α^−/−^ cells. Equal amounts (30 μg) of protein extracts from HepG2 (*Nrf1α*^+*/*+^) and HEA157 (*Nrf1α*^−*/*−^) cells were subjected to electropherotic separation by SDS-PAGE containing 8% or 10% polyacrylamide. Subsequently, the resolved proteins were determined by immunoblotting with distinct primary and secondary antibodies, followed by visualization by using the enhanced chemiluminescence. The intensity of immunoblotted protein bands was quantified by using the Quantity One software developed at Bio-Rad Laboratories, and normalized to the levels of β-actin as an internal control to verify the amount of proteins loaded in each well. The results were calculated as a fold change (mean ± S.D) of protein expression levels of genes controlling the cell cycle (**a**), metastatic and invasive behaviour including the EMT markers (**b**) in *Nrf1α*^−*/*−^ cells, and are also shown graphically (**c**,**d**) herein as a representative of at least three independent experiments undertaken on separate occasions. Significant decreases (*p < 0.05, **p < 0.01, n = 9) or significant increases (^$^p < 0.05, ^$$^p < 0.01, n = 9) are indicated, relative to corresponding protein levels of indicated genes expression in wild-type *Nrf1α*^+*/*+^ cells.

For a mechanistic insight into the above alterations in the cell cycle and apoptosis, herein quantitative real-time PCR and western blotting were performed to examine whether loss of Nrf1α results in dyregulation of key genes crucial for the cell cycle control. As anticipated, the expression of cyclin-dependent kinase 2 (CDK2, a marker of controlling the cell cycle progression from the S to G2 phases) was significantly down-regulated by knockout of Nrf1α at basal levels of mRNA (Fig. 12a) and protein (Fig. 13a2) expression, when compared with equivalent values obtained from *Nrf1*^+*/*+^ control cells. The finding supports the notion that down-regulation of CDK2 leads to a reduction in the S-phase of *Nrf1α*^−*/*−^ cells (Fig. 11a,b). The S-phase reduction is also attributable to down-regulation of cyclin-dependent kinase inhibitor 1A of p21 (i.e. p21^CDKN1A^, Figs 12a and 13a7), which resulted from impaired expression of its upstream p53, a conserved tumour repressor controlling cell cycle division and progression (Fig. 13a6). In addition, down-regulation of the p53-p21-CDK2 signaling pathway by *Nrf1α*^−*/*−^ may also lead to other alterations in the cell cycle (e.g. the G2-M arrest as described in Fig. 11a,b).

Intriguingly, knockout of Nrf1α up-regulated the mRNA expression of genes encoding cyclin-dependent kinase 6 (CDK6) and Cyclin D1 (both are involved in a functional complex controlling the G1-S transition) in *Nrf1α*^−*/*−^ HEA157 cells (Fig. 12a). Consistently, the abundance of the entire CDK6 protein was increased (Fig. 13a3), as accompanied by a modest increase in the active fraction of T286-phosphorylated Cyclin D1 (Fig.13a4), albeit the non-phosphorylated Cyclin D1 protein was obviously decreased (Fig. 13a5); this occurs possibly because the non-phosphorylated protein is unstable to be allowed for rapid degradation as described elsewhere^75^. In addition to CDK6, its cognate inhibitor p16 cyclin-dependent kinase inhibitor 2A (i.e. p16^CDKN2A^, which is involved in the restriction control within the G1 phase to enter either the S phase or undergo cell senescence) was up-regulated in *Nrf1α*^−*/*−^ cells (Figs 12a and 13a8). The G1-S progression of the cell division cycle was also limited by increased expression of retinoblastoma protein 1 (Rb1, Fig. 13a9), which acts as a tumour repressor because it binds and inhibits the transcription activating complexes of E2 promoter-binding–protein-dimerization partners (E2F-DP) insomuch as to restrict the *Nrf1α*^−*/*−^ cells to enter the S phase, as described elsewhere^76^.

In addition, paradoxical dyregulation of genes involved in pro-apoptosis (e.g. Caspases 3, 4 and 6) and anti-apoptosis (e.g. Bcl2 and Bcl2l1) was also found in *Nrf1α*^−*/*−^ cells (Fig. 12b). However, the results cannot provide a better explanation of why *Nrf1α*^−*/*−^ cells display no changes in the cell cycle G0/G1 phase and the relevant early apoptosis, and hence this warrants the further mechanistic study.

### Deficiency of Nrf1α results in dysregulation of genes controlling cell shape and behaviour

Collectively, the aforementioned results demonstrate that loss of Nrf1α leads to marked phenotypic changes in cell shape and behaviour, such as migration, invasion, transformation, tumourigenesis and malgrowth of the carcinoma xenografts derived from *Nrf1α*^−*/*−^ HEA157 cells. For an in-depth insight into which subsets of genes controlling cell process and behaviour are dysregulated in Nrf1α^−/−^ cells, we carried out quantitative real-time PCR and western blotting in order to determine whether the constitutive expression of such genes at both mRNA and protein levels was impaired in *Nrf1α*^−*/*−^ cells. As anticipated, the results revealed down-regulation of critical genes encoding the epithelial marker proteins E-cadherin (CDH1) and cadherin-associated protein α1 (α-catenin, CTNNA1) in *Nrf1α*^−*/*−^ cells (Figs 12c and 13b3,b7). The down-regulation of such epithelial proteins was accompanied by up-regulation of key genes encoding the mesenchymal marker proteins N-cadherin (CDH2), vimentin and fibronectin 1 (FN1) (Figs 12c and 13b4–b6). Moreover, expression of α-Catenin and β-Catenin (both act as linking proteins between cadherins and actin-containing filaments of the cytoskeleton) was dysregulated in *Nrf1α*^−*/*−^ cells (Figs 12c and 13b). Together, these data indicate that loss of Nrf1α promotes the EMT process entailing a risk of malignant cancer behaviour, because the above genes are also involved in controlling the cytoskeleton deformation, cell migration and invasion.

The putative EMT appeared to be monitored by impaired expression of two zinc finger transcription factors Snail1 (SNAI1) and SNAI2 in *Nrf1α*^−*/*−^ cells (Figs 12c and 13b1,2), because both factors have been shown to act as key mediators of EMT through regulating expression of the target genes *CDH1* and *CDH2* by binding the E-box in their promoter regions, particularly in metastatic hepatocellular carcinoma^77^,^78^. Nrf1α knockout resulted in down-regulation of SNAI1 but up-regulation of SNAI2 at mRNA and protein levels (Figs 12c and 13b1,2), albeit the underlying mechanism is unknown. Moreover, loss of Nrf1α also up-regulated transcriptional (and translational) expression of genes encoding matrix metallopeptidase 9 (MMP9) and membrane-type MMP17 in *Nrf1α*^−*/*−^ cells (Figs 12c and 13b8); both were hence postulated to increase the breakdown of both extracellular matrix proteins (and pro-proteins) between cells insomuch as to induce cancer cell growth, invasion and metastasis, as described by^79^.

### Similar and different expression patterns of genes in between Nrf1α^−/−^ cells and derived xenografts

In terms of elevated migratory and invasive activity of *Nrf1α*^−*/*−^ cells through transwells (Fig. 7a–d), a lot of obvious metastatic tumours were also examined by histopathology of livers rather than other organs in the tumour-bearing mice injected subcutaneously with the Nrf1α knockout cells (Fig. 9a,b). Together with other data (Figs 12c and 13b), our evidence indicates that the *Nrf1α*^−*/*−^-promoted EMT is required for hepatic metastasis from *in situ* subcutaneous carcinoma location to livers. Next, changes in the *in vivo* expression levels of such genes in the xenograft carcinomas were examined. As expected, we found similarity in between *in vivo* (i.e. xenograft) and *in vitro* (i.e. cultured cells) expression patterns of most genes, which are involved in the EMT process as well as signaling pathways responsible for cell-cycle controls and cell apoptosis (Figs S5 and 12c). This finding is revealed by comparison of the real-time qPCR results obtained from xenograft (Fig. S5 showing several gene expression data calculated as a fold-regulatory change in relative mRNA levels compared to the respective internal control values) and cultured cells (Fig. 12 showing each of indicated mRNA expression data re-evaluated by normalization relative to corresponding wild-type values being defined as 1).

Intriguingly, almost no changes in the expression of vimentin (VIM, which acts as one of major mesenchymal markers) were observed, but this was instead accompanied by modestly increased abundance of the epithelial marker E-cadherin-associated α-catenin, in *Nrf1α*^−*/*−^-derived xenograft tumours (Fig. S5a). This observation appears contrary to the data obtained from original *Nrf1α*^−*/*−^ cells (Figs. 12c). As such, our other evidence that had been provided (Figs 12, 13 and S5a) still revealed that the putative EMT process is required for *in vivo* metastasis of the subcutaneous carcinoma xenograft to the livers of mice injected with Nrf1α knockout cells, but the detailed mechanisms remains to be further determined.

In contrast with *in vitro* expression patterns of genes involved in the cell cycle controls (Fig. 12a), CDK6 appeared to be unaltered by knockout of Nrf1α *in vivo* (Fig. S5b). Contrary to *in vitro* expression of Cyclin D1 (CCND1), an obvious decrease in its *in vivo* expression was examined in *Nrf1α*^−*/*−^ xenograft tumours (*cf*. Figs S5 with 12a). Albeit the relevance to xenograft malgrowth is unknown, the data (as shown in Fig. S5c) suggest that this malgrowth appears to be pertinent to anti-apoptosis up-regulated at Bcl2, as accompanied by pro-apoptosis down-regulated at Caspases 3 or 6.

### Disturbed expression of Nrf1α in the human hepatocellular carcinoma cell lines and tissues

**Figure 14:**
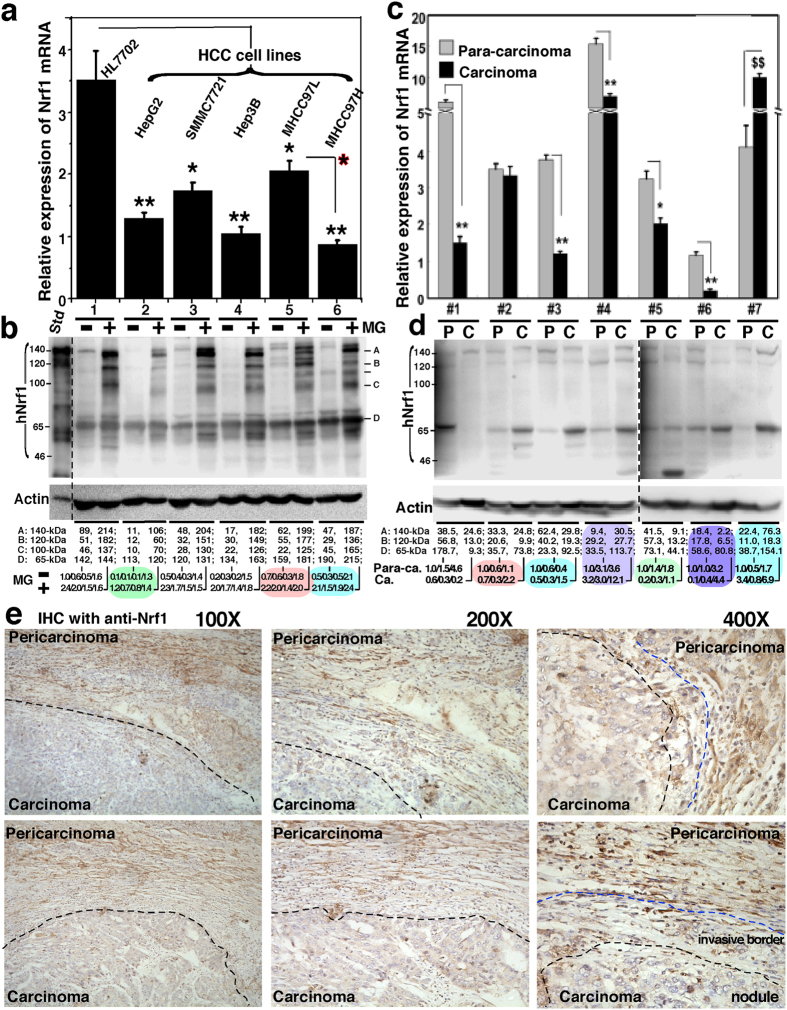
Disturbed expression of the constitutive Nrf1 mRNA and proteins in the human HCC and relevant cell lines. (**a**) Total RNAs were isolated from five distinct HCC cell lines together with the non-cancerous HL7702 cells (all without MG132) and then subjected to RT-qPCR of Nrf1, as described in Fig. 12. The results were calculated as a fold change (mean ± S.E) of Nrf1 in these samples, relative to their respective internal control levels. Significant decreases (*p < 0.05, **p < 0.01, n = 9) of Nrf1 mRNA expression in HCC cells are indicated as compared to the level measured from HL7702 cells. (**b**) Total lysates (30 μg of proteins) of the cells that had been treated with or without MG132, were subjected to electropherotic separation by 8% SDS-PAGE, followed by western blotting with anti-Nrf1 antibodies. The intensity of immunoblots of Nrf1α (and its derivates) and Nrf1β was quantified as described in Fig. 13, with a ratio of ~140-kDa, ~120-kDa, ~100-kDa and ~65-kDa proteins being calculated by normalization to the 140-kDa value measured from HL7702 cells. (**c**) Total RNAs were isolated from all seven pairs of the human carcinoma and para-carcinoma tissues that had been confirmed by histopathological examinations (see Fig. S6). The results of RT-qPCR were calculated as a fold change (mean ± S.E) of Nrf1 in these paired samples, relative to their respective internal control levels. Significant decreases (*p < 0.05, **p < 0.01, n = 9) or significant increases (^$^p < 0.05, ^$$^p < 0.01, n = 9) of Nrf1 mRNA expression in the HCC samples are determined by comparison to its levels measured from corresponding para-carcinoma tissues. (**d**) Equal amounts (50 μg) of protein extracts from the above carcinoma and para-carcinoma tissues were analyzed by western blotting with anti-Nrf1 antibodies. The intensity of immunoblots was quantified and the results are shown on the bottom with a ratio of ~140-kDa, ~120-kDa and ~65-kDa proteins in each sample (*cf*. para-carcinoma with carcinoma). (**e**) Two human HCC samples were visualized by immunohistochemistry with purified anti-Nrf1 antibodies. The areas of carcinoma nodules, invasive borders and pericarcinoma were roughly illustrated in the images that are a representative of at least three independent experiments undertaken on separate occasions.

The study of xenograft model mice raises a question of whether the endogenous expression of Nrf1α (or other isoforms) is down-regulated in the human hepatocellular carcinomas (HCC). To address this, we have performed the following experiments to determine whether the constitutive expression of human Nrf1α (and its derivates) is disturbed in HCC cell lines and tissues (that were removed surgically). As anticipated, total mRNA expression levels of Nrf1 (including all variants of its transcripts), together with basal and MG132-stimulated amounts of Nrf1α (and its derivates between 140-kDa and 85-kDa), but not of 65-kDa Nrf1β, are significantly diminished to lower levels detected in all five lines of HCC cells than those obtained from the non-cancerous HL7702 cells (Fig. 14a,b). By close comparison of the data obtained from MHCC97H and MHCC97L (both are known to have high and low metastatic potentials, respectively), it was found that their potential metastatic activities are negatively correlated with the extents to which Nrf1 mRNA and Nrf1α protein (and its derivates) are constitutively expressed (Fig. 14a,b; *cf. columns and lanes 5 with 6*). In other words, mRNAs and proteins of Nrf1 were expressed at modestly higher levels in the low metastatic MHCC97L cells only than those measured in the high metastatic MHCC97H cells.

Subsequently, similarly but differently disturbed expression patterns of Nrf1α (and its derivates), but not of Nrf1β, were examined in seven patients with distinct pathological severity of HCC (Figs 14c,d and S6). The real-time qPCR analysis revealed a markedly low abundance of total Nrf1 mRNA in poorly low-differentiated hepatocellular carcinoma (i.e. *C, sampled from the carcinoma nodules*), with tumour embolus being in vessels, and its para-carcinoma tissue (i.e. *P, sampled within a more than 2-cm distance from the carcinoma nodules*), and much lower level of mRNA expressed in the carcinoma compared to para-carcinoma (Fig. 14c, *column* #6, and S6f). Western blotting of low-differentiated carcinoma samples indicated a significant shortage of 140-kDa Nrf1α and its derived 120-kDa proteins, when compared with their expression levels in relevant para-carcinoma tissues (Fig. 14d, *lanes* #6 *C vs P*).

In a sharp contrast with the poorly-differentiated HCC, highly well-differentiated hepatocellular carcinoma appeared to have considerably sufficient expression of Nrf1 mRNA (Figs 14c, *column* #7 and S6g), as well as Nrf1α with a slightly faster electrophoretic mobility to ~130-kDa estimated on 8% SDS-PAGE gels, when compared with that of the 140-kDa Nrf1α expressed in the para-carcinoma tissue (Fig. 14d, *lanes* #7 C vs P). Intriguingly, similar expression patterns of Nrf1 were not found in the high-to-medium differentiated HCC concomitantly with focal necrosis (Figs 14c, *column* #2, and S6b), but the complex lesions appear to be associated with disturbed expression of the putative active ~120-kDa Nrf1α protein (Fig. 14d, *lanes* #2 C vs P).

By comparison of the data obtained from HCC samples #*3* and #*2* (Figs 14c,d and S6c), blunted expression of Nrf1 mRNAs, but neither the 140-kDa nor 120-kDa Nrf1α proteins, was indeed suggested to be relevant to additional lesion of cirrhosis. The notion is also supported by altered Nrf1 expression in the para-carcinomas suffered from cirrhosis or not (Figs 14c, *cf. columns* #1 with #5 and S6a with S6e). Further comparisons of results measured from HCC samples #1, #5 and #7 revealed that expression of Nrf1 mRNA and its products (particularly of ~120-kDa Nrf1α) is significantly decreased in intermediately-differentiated carcinomas compared to its para-carcinoma tissue (Figs 14c,d and S6a,e,g).

Collectively, the extent of disturbed expression of constitutive Nrf1 mRNA and Nrf1α protein (and/or its derivates of between 140-kDa and 100-kDa) is postulated to be relevant to the pathological severity of HCC with distinct differentiation and metastatic potentials, together with additional concomitant lesions (e.g. inflammation, necrosis, fibrosis and cirrhosis). However, an eccentric exception is that a medium-to-low differentiated hepatocellular carcinoma (with tumour embolism being in vessels) seemed to give rise to an unexpectedly incremental expression level of Nrf1 mRNA, but not of Nrf1α proteins (Figs 14c,d cf. #4 with #6, & S6d vs f). Although this unusual observation remains to be further clarified, it cannot be ruled out that some error-sampled tissues were contaminated during manipulation by practical surgeons with the naked eyes to hardly distinguish the cancerous nodules (and embolus) from cirrhotic nodules. For this reason, relevant HCC tissues were further subjected to immunohistochemistry of HCC with antibodies against Nrf1. The results demonstrated a gradient staining pattern of Nrf1 radially from the core carcinoma nodules towards the pericarcinoma tissues (Fig. 14e). Much less or none of the immunoreactive Nrf1-staining was seen in the center of cancerous nodules, whilst a fainter staining was in the putative invasive borders between the carcinoma nodules and pericarcinoma surroundings, as accompanied by a relatively stronger staining in the pericarcinoma tissues (Fig. 14e, *lower panels*). However, a weak point of the anti-Nrf1 immunohistochemistry of HCC should be noted that it does not serve to distinguish Nrf1α (and/or its derivates) from Nrf1β. This is owing to the fact that the 65-kDa Nrf1β, but not Nrf1α (or its derivates), was determined to exist in the carcinoma to a relatively higher degree than that expressed in the para-carcinoma (Fig. 14d), although whether Nrf1β exerts a specific effect on the HCC pathology remains elusive. Lastly, not any mutations in the primary amino acids of Nrf1α was found by its cDNA sequencing of HCC cell lines and relevant carcinoma tissues (data not shown herein).

## Discussion

In the present study we have established the human homozygous Nrf1α knockout cells and hence discovered that: i) the resulting *Nrf1α*^−*/*−^ cells exhibit obvious morphological phenotypes with the cell-cycle alterations, which are distinct from the wild-type (*Nrf1*^+*/*+^) parent hepatoma cells; ii) loss of Nrf1α leads to significant increases in the cell proliferation, invasion, migration, transformation, carcinogenesis and malgrowth when compared with the control values; iii) loss of Nrf1α’s function results in dysregulation of key genes controlling cell process and behaviour; iv) the putative EMT process is promoted by knockout of Nrf1α, leading to hepatic metastasis in the xenograft model mice that had been injected subcutaneously with *Nrf1α*^−*/*−^-derived cancer cells; and v) the extent to which constitutive expression of Nrf1 mRNA and particularly Nrf1α (and its derivative) proteins is markedly disturbed in the human hepatocellular carcinoma (HCC) cell lines and tissues is of paramount relevance to the pathological severity of the malignant disease.

In the past two decades, genome editing of *Nrf1* (also called *nfe2l1*) in the mouse and relevant cell lineages was primarily achieved *via* the introduction of donor DNA targeting vectors that contained homologous sequences to the gene locus and also distinct exogenous DNA fragments for knockout^30^,^80^, knock-in^31^, or Cre-LoxP strategies^33^,^34^,^35^. The ensuing homologous recombination between the donor DNA vector and the cognate gene was allowed for desirable disruption of the genomic *Nrf1* sequence containing the codons of its DNA-binding domain within almost all isoforms (e.g. Nrf1α, LCR-F1/Nrf1β, Nrf1γ, and Nrf1δ)^42^,^43^,^44^. Although these gene-targeting strategies have proven invaluable in studies of the gene structure and function, the homologous recombination process works very inefficiently in mammalian cells (particularly in the human, as reviewed by^81^), such that none of Nrf1 isoform-specific knockout cell lines have been established before herein. Therefore, it is unknown which isoforms of Nrf1 contribute to the significant pathological phenotypes of different model mice that had been created in the above gene-targeting experiments.

Recently, it was found that the efficiency of gene editing *via* homologous recombination is increased by the introduction of double-strand breaks into DNA of target genes that is directed by engineered nucleases, in the presence of suitable donor DNA sequences^82^. Since targeted double-strand breaks are easily introduced into the site-specific DNA by using TALENs, the combination of this technology with the transfection of a homologous donor DNA repair template has become a popular approach to enable the precise manipulation of mammalian genome^83^. Herein, TALENs-mediated editing of the human genomic *Nrf1* sequence has been carried out insofar as to create knockout of full-length Nrf1α-specific isoform, rather than other smaller LCR-F1/Nrf1β, Nrf1γ and Nrf1δ forms, in the hepatocellular carcinoma HepG2 cells, followed by establishment of a stable monoclonal cell line with the homozygous *Nrf1α*^−*/*−^ deletion mutations.

Consequently, it was found that loss of Nrf1α leads to obvious phenotypic changes in the morphology of HepG2-derived *Nrf1α*^−*/*−^ cells, which become elongated within slender spindle-like or triangle-protruded shapes. However, the host epithelial surface structures disappear from the smooth surface of Nrf1α^−*/*−^ cells, so that these cell-cell interaction gaps are enlarged. These alterations in morphological phenotypes of between Nrf1α^−*/*−^ and Nrf1^+*/*+^ cell lines have led us to suppose that Nrf1α-specific knockout enables the cells to undergo the putative EMT, a process entailing a risk of cancer transformation. Further evidence that has been presented demonstrates that knockout of Nrf1α results in significant increases in both the invasive and migratory abilities of *Nrf1α*^−*/*−^ cells, insomuch as to have promoted the colony formation of such cells grown on soft agar and *in vivo* malgrowth of relevant subcutaneous carcinoma xenograft in immunodeficient nude mice. The worsening consequences are thus inferred to result from loss of Nrf1α’s function as a potential repressor to confer on the host cytoprotection against cancer cell proliferation, malignant transformation and carcinogenesis. This notion is further supported by molecular expression results revealing that knockout of Nrf1α results in dysregulation of key genes involved in the cell process, cytoskeleton deformation and the putative EMT dedifferentiation. This suggests a possible gene regulatory networking mechanism leading to deterioration of *Nrf1α*^−*/*−^ hepatoma cell behaviour (i.e. invasion, migration and malgrowth).

Fluorescence-activated sorting of *Nrf1α*^−*/*−^ and *Nrf1*^+*/*+^ hepatoma cells revealed that Nrf1α knockout causes the cell cycle to be arrested at the G2-M phase along with the S-phase being reduced. This is accompanied by a modest decrease in Nrf1α-deficient cell apoptosis occurring at the later, rather than the early, stages. For a mechanistic insight into the cell-cycle alterations that facilitate *Nrf1α*^−*/*−^ cell division and proliferation, basal expression levels of key genes were further determined after a high-throughput measure of gene expression profiling to create a global structure of cellular function. Of note, it was found that loss of Nrf1α’s function results in dysregulated expression of genes critical for the cell-cycle control. In addition, it is intriguing to note that the basal expression of some genes involved in pro-apoptosis or anti-apoptosis is dysregulated in *Nrf1α*^−*/*−^ cells, but the controversial events remain to be further determined.

Taken together, our results demonstrate that loss of Nrf1α (and its processed products of between 140-kDa and 100-kDa), but not of LCR-F1/Nrf1β, Nrf1γ or Nrf1δ, in the human hepatoma HepG2 cell line contributes deterioration of the resulting *Nrf1α*^−*/*−^ cells in relevant process and behavour. The Nrf1α-deficient cell platform has been established insofar as to provide a better understanding of the human transcription factor that acts as a potential tumour repressor to monitor the homeostatic expression of cytoprotective genes against cancer development and its malignant behaviour (further data from cDNA sequencing not shown herein).

More interestingly, we have provided the evidence showing that the putative EMT process is promoted as the unique function of Nrf1α is lost in the hepatoma cells, and that the hepatic metastasis occurs in the xenograft model mice injected subcutaneously with *Nrf1α*^−*/*−^, rather than wild-type *Nrf1α*^+*/*+^, HCC cells. Further evidence that has been obtained from cell biological and molecular studies of cultured cells (i.e. *in vitro*) and xenograft tumours (i.e. *in vivo*) supports the notion that the escape of HCC cells from the solid tumour foci *in situ* is due to dedifferentiation of the epithelial cells, as it occurs by loss of cell-to-cell contacts (i.e. cell adhesive junctions) and instead, the concomitant gain of migratory and invasive abilities with which the mesenchymal cells are intrinsically endowed. The phenotypic shift of cells is designated as the EMT^84^,^85^, a cellular program that is defined (refs 86, 87, 88 and in this study) by three major changes in the phenotype: i) the morphology converted from a cobblestone-like epithelial cells with an apical-basal polarity to dispersed, spindle-shaped mesenchymal cells with migratory protrusions; ii) differentiation markers switched from cell-cell junction proteins and cytokeratin intermediate filaments to vimentin filaments and fibronectin; and iii) the functional behaviour that accompanies the putative conversion from the immotile to motile cells; such activity is acquired for the motile cells to invade through the underlying extracellular matrix and migrate to suitable sites (i.e. a functional hallmark of EMT).

**Figure 15:**
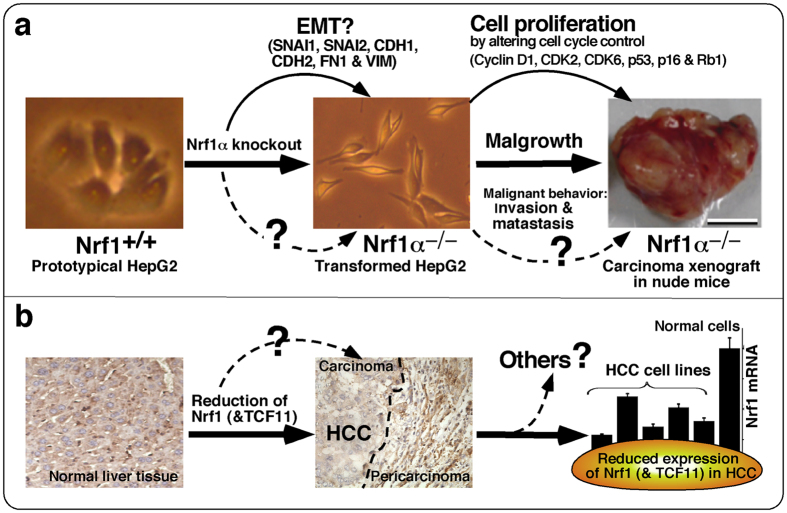
Schematic modeling of *Nrf1*α^−/−^-promoted EMT and other cell processes in the liver cancer development. (**a**) Schematic representation of Nrf1α^−/−^-promoted EMT and other cell processes involved in the mouse xenograft model. The wild-type (i.e. *Nrf1α*^+*/*+^) epithelial-like HepG2 cells are subjected to TALENs-directed site-specific knockout of Nrf1α in order to yield the resultant bi-allelic mutant (i.e. *Nrf1α*^−*/*−^) cells that are endowed with the mesenchymal-like characteristics. The phenotypic change is regarded as the epithelial-mesenchymal transient (EMT, which occurs at the primary tumour foci), whilst EMT is reversible to the process being referred to as the mesenchymal-epithelial transient (MET, which takes place at suitable metastatic sites). The putative EMT dedifferentiation is promoted to acquire the high-metastatic potentials following Nrf1α-specific knockout, as is accompanied by enhanced expression of Nrf1β, in the hepatocellular carcinoma HepG2 cells and relevant xenograft model mice. (**b**) The human constitutive expression of Nrf1α, but not of Nrf1β, is markedly attenuated or even abolished in the low-differentiated high-metastatic hepatocellular carcinoma, amongst a series of paired carcinomas nodules and surrounding (pericarcinoma or para-carcinoma) tissues. The hepatic Nrf1α expression is further deteriorated by chronic (uncontrollable) inflammatory, fibrotic and cirrhotic lesions. Collectively, our evidence that has been presented (and some data not shown) in the paper, together with previous studies revealing that potential switches between EMT and MET control the hepatocarcinogenesis and its malignant progression (i.e. transformation, malgrowth, invasion and metastasis), has let us to propose that Nrf1α (but not Nrf1β) might monitor such EMT-MET switches to repress hepatic cancer development. The notion is also supported by the fact that the putative functional loss of Nrf1α (though Nrf1β/γ are also lost) in the mouse liver results in the hepatic spontaneous cancer^33^,^34^. Overall, Nrf1α is of significant importance in the physio-pathological origin and development, but further studies are warranted to address a new open question of whether (and how) Nrf1α monitors the putative EMT-MET reprogramming during the embryonic and cancer development.

Together with our data presented herein, it is thereby proposed that the *Nrf1α*^−*/*−^-promoted EMT causes the aberrant proliferation and dedifferentiation of malignant hepatocytes to possess increased migratory phenotype insomuch as to play a pivotal role in the dissemination, invasion and metastasis of the Nrf1α-deficient hepatoma cells during tumour progression of HCC (that has malgrown and outgrown possibly through mechanisms by which the cell division cycle and anti-apoptosis are enhanced by loss of Nrf1α) in the xenograft model mice (Fig. 15a). Such deterioration was, in large part, rescued by restoring ectopic wild-type Nrf1α into the *Nrf1α*^−*/*−^ hepatoma cells (data not shown), suggesting that it may exert an anti-cancer preventive effect against HCC heterotransplanted in the xenograft nude mice. This assumption is also supported by further molecular pathology of the human HCC, revealing a marked decrease or abolishment in the constitutive expression of Nrf1 mRNA and particularly Nrf1α (and/or its derivatives of between 140-kDa and 100-kDa), but not Nrf1β/γ, proteins examined in the carcinoma tissues and relevant cell lines. By close comparison of MHCC97L and MHCC97H, it is found that their potentials to elicit a low or high metastatic activity are negatively correlated with the extent to which Nrf1 mRNA and its product Nrf1α (and its derivates) are constitutively expressed. This is to say that both Nrf1 mRNA and Nrf1α protein were at marginally higher levels measured in the low metastatic MHCC97L cells, when compared to those being significantly attenuated or even abolished in the high metastatic MHCC97H cells. Moreover, the extent of disturbed expression of the constitutive Nrf1α (and its derivates), rather than Nrf1β/γ, is also determined to be pertinent to the pathological severity of HCC with distinct differentiation and metastatic potentials, together with additional concomitant lesions (e.g. chronic inflammation, fibrosis, cirrhosis and necrosis). Collectively, it is inferable that Nrf1α might act as a reprogramming repressor of EMT to maintain the epithelial integrity and thus suppress its dedifferentiation, such that the homeostatic Nrf1α is conferred on the host to possess an intrinsic anti-cancer preventive effect against carcinogenesis and tumour progression, albeit the detailed molecular mechanisms remain to be elucidated.

Conversely, loss of the putative Nrf1α‘s function is proposed to be a major (or essential) contributor leading to spontaneous development of hepatoma in liver-specific *Nrf1*^−*/*−^ mice (in which Nrf1β/γ, besides Nrf1α, is also deleted). The hepatic carcinogenesis was originally thought to result primarily from the severe oxidative stress-induced NASH with disordered lipid metabolisms^33^,^34^. Nonetheless, whether (or how) Nrf1β/γ contributes to cancer development is unknown, whilst the constitutive abundance of Nrf1β appears to be increased, relative to Nrf1α, in the human HCC, particularly poorly low-differentiated carcinomas, as compared to the para-carcinoma tissues sampled from the same patients. Similarly, a major endogenous protein of Nrf1β, but neither Nrf1α nor its derivates of between 140-kDa and 100-kDa, was detectable in human erythroleukemia (K562) cells^45^. These raise a possibility that the relatively increased Nrf1β, as being contrary to Nrf1α, is involved in cell transformaition, carcinogenesis and cancer progression, probably through promotion of the putative EMT process.

Notably, the reprogramming of EMT is determined to be the basis indispensable for the formation of a three-layer embryo, a process named gastrulation^89^,^90^,^91^, and certain switches between EMT (epithelial-mesenchymal transition) and MET (mesenchymal-epithelial transition) are defined as a remarkable feature of gastrulation during early embryonic development^90^,^92^,^93^. As such, it is intriguing that the failure to form the primitive steak mesoderm, although ectoderm and visceral endoderm layers appeared normal, causes the embryonic lethality in the early gastrulation (between 6.5 and 7.5 dpc) of global knockout mice with a deletion of a 3.5-kb genomic nucleotide sequence encompassing aa 172-741 of Nrf1, such that all isoforms including Nrf1α and Nrf1β/γ were lost completely^30^. However, the non-cell-autonomous defect had been not emerged in additional global knock-in mutant mice that died at mid-late gestation from 13.5 to 18.5 dpc^31^. The latter phenotype may be affected by remaining expression of residual Nrf1 isoforms of between 120-kDa and 36-kDa, which had been retained to varying extents^31^,^32^,^94^,^95^,^96^. Therefore, we speculate that Nrf1α and Nrf1β (and their derivates) might be involved in the transcriptional regulation of some critical genes responsible for controlling mesodermal formation, but whether or how they might monitor the potential switches between EMT and MET during embryonic development is a new open question to be addressed.

Significantly, the putative EMT-MET reprogramming occurs at the crossroads of development and carcinogenesis, as well as cancer progression and metastasis^88^,^93^,^97^,^98^. When hijacked during the development of cancer, such as HCC^90^,^93^,^97^, the cells to undergo EMT are acquired for an ability to leave the primary tumour, some of which re-undergo MET at secondary sites and thus are enabled for devastating consequences on the host, allowing tumour cells derived from epithelia to invade surrounding tissues and spread through the organism.

The cellular origin of HCC is represented by hepatocytes or even progenitor cells of the liver^99^,^100^. The epithelial hepatocytes are maintained at mitotic inactive states under normal physiological conditions and are also enabled for high differentiation only to meet the requirement of their specialized functions for glucose, amino acid and lipid metabolism, as well as detoxification. Under pathophysiological conditions, hepatocytes display an vastly extraordinary proliferative capacity for regenerative and non-regenerative repairs in response to damaging stimuli^101^, part of which include chronic and/or malignant lesions resulting from viral infection, intoxication, inflammation, fibrosis, cirrhosis, and other carcinogenic insults insomuch as to acquire initial genomic alterations which are further accumulated during hepatocarcinogenesis^99^,^102^. Of note, the reprogramming of EMT is actively involved in the fibroproliferative wound healing known as fibrosis^100^; this is associated with chronic inflammatory (e.g. NASH) and cirrhotic lesions that commonly precede the dysplastic HCC-like foci and nodules^103^. Moreover, the putative switches between EMT and MET were reported under control of the tumour microenvironments during the HCC development and progression^104^. Collectively, targeting EMT (or switches between EMT and MET) could be paved as a novel strategy to combat the human HCC. Together with the aforementioned anti-cancer preventive effect of Nrf1α to repress the EMT process, we thus propose that activation of the full-length CNC-bZIP transcription factor (and its derivates) could be developed as a putative chemopreventive target to defense against HCC, albeit it remains to determine the detailed mechanisms by which disturbed expression of the intact protein (and its derivates) leads to the disease pathogenesis and malignant progression (Fig. 15b).

## Materials and Methods

### Chemicals and antibodies

All chemicals were of the highest quality commercially available. The proteasome inhibitor MG132 was purchased from Sigma-Aldrich and used herein at final concentration of 5 μmol/L. The anti-sera against Nrf1 that were developed in rabbits using a polypeptide covering amino acids 292–741 in our own laboratory, whilst another anti-Nrf1 antibody, as well as an specific antibody against phosphorylated Cyclin D at Thr^286^, was purchased from Cell Signaling Technology, Inc (branched in Shanghai, China). Rabbit polyclonal antibodies against MMP9, SNAI1, p16 and Vimentin were bought from BioSynthesis (Beijing, China), whilst other rabbit antibodies against α-Catenin, CDK2, CDK6, Cyclin D1, SNAI2, FN1, Rb1, p53 and p21 were purchased from BosTer (Wuhan, China). Mouse monoclonal antibodies against CDH1 and CDH2 were from ABGENT Ltd (Suzhou, China). Anti-β-Actin antibody was from Zhongshan Jinqiao Co (Beijing, China). In addition, ER/DsRed (an ER-localized red fluorescent protein marker) was obtained from BD Biosciences (USA).

### Cell culture and transfection

Both cell lines of the human hepatocellular carcinoma (HepG2) and human embryonic kidney (HEK293) were originated from ATCC (Zhong Yuan Ltd., Beijing, China), and have been permanently maintained in our laboratory. These cells were grown in DMEM supplemented with 5 mmol/L glutamine, 10% (v/v) foetal bovine serum (FBS), 100 units/ml of either of penicillin and streptomycin, in the 37 °C incubator with 5% CO_2_. The experimental cells were transfected for 6 h with a reagent called FuGENE^®^ HD (Promega) and then allowed for recovery from transfection in the fresh medium for 12 h before being subjected to indicated experiments. Immortalized non-cancerous HL7702 cells and other HCC cell lines including SMMC7721, Hep3B, MHCC97L and MHCC97H were also originated from ATCC.

### Expression constructs for TALENs-mediated editing of the human *Nrf1* gene

Two expression constructs for TALENs (called TALEN-Left and TALEN-Right, both are required for genome editing of the human *Nrf1* gene sequence) were made by one-step ligation, using the FastTALE^TM^ TALEN assembly kit (from SIDANSAI, Shanghai, China). Their target sequences for DNA-binding domains (DBD) of TALENs to the human *Nrf1* gene were designed: the left arm recognizes 5 ′-TAAACATTCTGGTCCT-3′, whilst the right arm recognizes 5′-TCCGTTAAGTATTTCTT-3′ (within both sequences, the first underlined T is a conserved base that has been positioned just 5′ to each of the target nucleotide fragments) (Fig. 2a,b). Between these two *Nrf1*-binding sites there is an 18-bp spacer (5′-TCAGCAATGCTTTCTCTG-3′, in which the underlined three bases ATG represent the start codon to translate Nrf1α). Subsequently, TALEN-left and TALEN-right plasmids were constructed according to the standard manipulation protocols provided by the manufacturer, followed by the sequencing of purified plasmids in order to confirm the fidelity of DBD-coding fragments inserted.

### Nrf1α-specific knockout cell line established by TALENs

To ensure the activity of TALENs, the human HEK293 cells (1 × 10^5^) that had been grown in 6-well plates were transfected for 6 h with a pair of expression constructs for TALEN-Left (1.5 μg) and TALEN-Right (3.0 μg) in a mixture with 15 μl of the reagent FuGENE^®^ HD at a ratio of 1:2:10 (w/w/v) before being allowed for recovery from transfection in the fresh medium for 12 h. The cells were harvested at 72 h after transfection, and were subjected to genomic DNA extraction and subsequent amplification by PCR (with a pair of primer: 5′-CGAGAAGGGAAA GTGAATG-3′ and 5′-CTGGGTCTGAGTATAGGCA-3′). These PCR products were cloned into the pMD19-T vector (Takara, Dalian, China) and then sequenced in order to identify whether (and which types of) the frameshift mutation occur in close proximity to TALENs-targeted sites.

To obtain Nrf1α-specific knockout cell line, the human HepG2 cells (1 × 10^5^) that had been grown in 6-well plates were transfected for 6 h with expression constructs for TALENs as described above. The cells were selected by addition of puromycin (2.5 μg/ml) to enable all the untransfected cells to be killed for 36–48 h, and thereafter were subjected to the single cell cloning, each clone of which was allowed for expansive growth to a certain extent in 96-well plates. Subsequently, the genomic DNA was extracted from these individual monoclonal cells and then amplified by PCR, followed by the sequencing of PCR products that were cloned into the pMD19-T vector. The sequencing results were analyzed by using the Clone Manager Suite.

### Quantitative real-time polymerase chain reactions (RT-qPCR)

Experimental cells (2 × 10^5^) that had been grown in 6-well plates were subjected to extraction of total RNAs by using an RNAsimple total RNA kit (Tiangen, Beijing, China), and then 1.5 μg of total RNA served as a template for subsequent synthesis of cDNA by using a RevertAid first strand cDNA synthesis kit (Thermo Fisher Scientific, USA), which were performed according to the manufacturer’s recommendations. The resulting cDNA products (15 ng) served as the templates of quantitative real-time PCR within 5 μl of the GoTaq^®^qPCR Master Mix (Promega, USA), qRT-PCR that was performed in the following conditions: activation at 95 °C for 30 s, followed by 40 cycles of 10 s at 95 °C, and 30 s at 60 °C. The mRNA of β-actin was used as an internal control, whereas a negative control was set in the reaction mixture without the cDNA templates. All the real-time PCR reactions were carried out in at least 3 independent experiments that were each performed triplicate. The results were analyzed by using the Origin 8.0 software. The sequences of the primers used in the study are shown in Table 1.

### Western blotting

Experimental cells (5 × 10^5^) were seeded in 35-mm dishes and cultured for 36 h before being harvested in the sample lysis buffer (2 mM Tris pH 7.5, 5 mM NaCl, 0.5 mM Na_2_EDTA, 0.04 mM DTT, 0.5% SDS) containing 2 μg/ml protease inhibitor cocktail (Roche, Germany). The clarified supernatants were collected and the protein concentrations were determined by using a BCA protein assay kit (Bi-Yuntian, Beijing, China) and boiled for 10 min. Then total cell lysates were subjected to the protein separation by Laemmli SDS-PAGE gels containing 8% or 10% polyacrylamide in the pH 8.9 Tris-glycine running buffer or by LDS-NuPAGE gels containing 4–12% polyacrylamide in the pH 7.3 MES running buffer. The protein-transferred PVDF membranes were blocked by incubation with 5% bovine serum albumin at room temperature for 30 min and then immunoblotted with each of the primary antibodies for overnight at 4 °C. Thereafter, the immunoblots were cross-reacted for 1 h with the corresponding species-specific secondary antibodies, followed by visualization by using either the enhanced chemiluminescence as described previously^105^,^106^. The intensity of blots was calculated by using the Quantity One software developed at Bio-Rad Laboratories, and normalized to protein-loading controls as described in figure legends.

### Immunocytochemistry and confocal microscopy

Experimental cells (2 × 10^5^) that had been allowed for overnight growth on a cover glass placed in 6-well plates were transfected for 6 h with an expression construct for the ER/DsRed marker protein and then were allowed for 12-h recovery from transfection in the fresh complete medium. The cells were fixed for 15 min with 4% paraformaldehyde in PBS buffer. Thereafter, the cells were permeabilized for 10 min with 0.1% Triton X-100 in PBS, before immunocytochemistry with the primary antibodies against Nrf1 (dilution 1:200) at 4 °C overnight. The immunostained cells were visualized after incubation with the DyLight 488 AffiniPure Goat anti-rabbit IgG (dilution 1:200, EarthOx, San Francisco, CA, USA) for 1 h at room temperature in the dark, followed by the nuclear DNA staining with DAPI for 5 min. The fluorescence images was observed and photographed under a confocal microscope (Leica, Germany) as described previously^59^,^106^.

### Scanning Electron Microscopy

Experimental cells (2 × 10^5^) were allowed for growth on the cover glass placed in 6-well plates overnight, before being fixed with 4% glutaraldehyde. The dried cell samples were gold-coated and then subjected to morphological observation under scanning electron microscope (S-3400N, Hitachi High-Technologies Corporation, Japan). In addition, these cells were also observed under general light microscopes (Olympus, Japan).

### The *in vitro* scratch assay

After experimental cells (1 × 10^5^) that had grown in 6-well plates reached 70% confluency, they were allowed for synchronization by 12-h starvation in a serum-free medium and then treated for 6 h with 1 μg/ml of mitomycin C (from Cayman, USA, an inhibitor to block genomic DNA replication in the cell division cycle). Subsequently, a clear ‘scratch’ in the cell monolayer was created and then allowed for being healed in the continuous culture at 37 °C with 5% CO_2_. The scratched images were captured at the beginning and at 12-h intervals during cell migration to close the scratch, followed by quantification of the cell migration as described elsewhere^68^.

### Transwell-based migration and invasion assays

Both transwell migration and invasion assays were performed in modified Boyden chambers (Transwell; Corning Inc. Lowell, MA, USA) as described previously^69^. Briefly, after the growing cells reached 70% confluency, they were starved for 12 h in a serum-free medium before being tripsinised. Then experimental cells (5 × 10^3^) were suspended in 0.5 ml medium containing 5% FBS and seeded in the upper chamber of a transwell, which allows the cells to grow on the microporous polycarbonate membrane that is tissue culture-treated to enhance cell attachment to the bottom, after migratory cells passed through the 8-μm microporous membrane. The cell-seeded transwells were placed in each well of 24-well plates containing 1 ml complete medium (i.e. the lower chamber), and then cultured for 24 h in the incubator at 37 °C with 5% CO_2_. Of note, the upper chamber bottom of transwells was pre-coated by matrigel basement matrix (BD, Biosciences, USA) before the cells were placed in the invasion assay. The remaining cells in the upper chamber were removed, and then the cells attached to the lower surface of the transwell membranes were fixed with 4% paraformaldehyde and stained with 1% crystal violet reagent before being counted.

### The soft agar colony formation assay

The anchorage-independent colony formation assay was performed according to the standard protocol^71^. Briefly, the cell culture plate (with a diameter of 100 mm) was coated by the basement gel containing 0.6% soft agar resolved in the pre-heated complete medium. The upper gel containing 0.3% soft agar and 1.25 × 10^4^ of experimental cells (that had been growing in the exponential phase) was allowed for two-layer gel formation on the above plate, before being cultured for 2–3 weeks in the incubator at 37 °C with 5% CO_2_. The cell clones formed on the soft agar plate were stained with 1% crystal violet reagent before being counted.

### Subcutaneous tumour xenografts in nude mice

Mouse xenograft models were made by subcutaneous heterotransplantation of the human hepatoma HepG2 or its derived Nrf1α-specific cells into nude mice as described^72^. Experimental human hepatoma cells (1 × 10^7^, that that had been growing in the exponential phase) were suspended in 0.2 ml of serum-free DMEM and were inoculated subcutaneously into the right upper back region of male nude mice (BALB/C^*nu/nu*^, 4–6 weeks, 18 g, from HFK Bioscience, Beijing) at a single site. The procedure of injection into all mice was completed within 30 min, and then formation of the subcutaneous tumour exnografts was observed. Once the tumour exnografts emerged, their sizes were successively measured once every other day, until six weeks when the mice were sacrificed before the transplanted tumors were excised. The sizes of growing tumours were calculated by a standard formulate (i.e. V = ab^2^/2) and then are shown graphically (n = 7 per group). The tumour tissues were subjected to the histopathological examination by the routine hematoxylin-eosin staining, followed by immunohistochemical staining with antibodies against Nrf1, E-cadherin (CDH1), and N-cadherin (CDH2). All mice were maintained under standard animal housing conditions with a 12-h dark cycle and allowed access *ad libitum* to sterilized water and diet. All relevant studies were carried out on 8-week-old male mice (with the personal licence No. PIL60/13167) in accordance with the United Kingdom Animal (Scientific Procedures) Act (1986) and the guidelines of the Animal Care and Use Committees of Chongqing University and the Third Military Medical University, both of which were subjected to the local ethical review. All relevant experimental protocols were approved by the University Laboratory Animal Welfare and Ethics Committee (with two institutional licenses SCXK-PLA-20120011 and SYXK-PLA-20120031). As for additional ethical concerns about the xenograft model mice bearing so big tumours insomuch as to give rise to certain bleeding ulcers, such a bad health condition of mice was only emerged from day 2 prior to being sacrificed, and the relevant study was indeed conducted according to the valid ethical regulations that have been approved.

### The human HCC tissue specimens

All seven human HCC are surgically sampled to be paired with the respective pathological adjacent liver tissues (i.e. para-carcinoma *sampled within a more than 2-cm distance from the carcinoma nodules*) from the Third Hospital Affiliated to the Third Military Medical University, Chongqing, China. The Informed Consent was given for the permission by each of the patients before all disease samples were histopathologically confirmed prior to RT-qPCR, western blotting and immunohistochemistry. All relevant protocols used in this study had been approved by the hospital’s Protection of Human Subjects Committee, and thus all experiments were carried out in accordance with the approved guidelines and related regulations.

### The immunohistochemistry of xenograft and human HCC tissues

All the paraformaldehyde (4%)-fixed and paraffin-embedded samples were sectioned into a series of 4-μm-thick slides, and then the slides were de-paraffinized in a solution of xylol and dehydrated in the concentration-graded ethanol before inactivation of endogenous peroxidase activity. Subsequently, the samples were allowed to be boiling in microwave for 15 min in a citrate buffer (pH 6.0) to retrieve antigen, and were then blocked with 1% bovine serum albumin for 60 min. The sampled sections were incubated overnight at 4 °C with the primary antibodies against Nrf1, CDH1, CDH2 and p16 (at their dilutions of 1:50, 1:100, 1:100 and 1:50, respectively). Thereafter, the primary antibody-staining slides were re-incubated with a biotin-conjugated secondary antibody for 60 min at room temperature, and visualized by the peroxidase-conjugated biotin-streptavidin complex (BosTer, Wuhan, China). In the similar experimental settings, the negative controls were set up by replacing the primary antibody with the normal non-immune serum diluted in PBS. The resultant images were obtained under a light microscope (Leica DMIRB, Leica, Germany) equipped with a DC350F digital camera, and the sample immunoreactivity was scored by the observer blinded to the identity of the samples.

### Recombinant lentivirus production

To create a stable Nrf1α-expressing hepatoma cell line, its lentiviral construct (Lenti-pEZ-Lv203), together with the GFP-expressing lentiviral control vector, was designed before being made by (GeneCopoeia, Guangzhou, China). Briefly, the lentiviral packaging 293T cells (1**×**10^6^) were seeded in a 10-cm dish and cultured in 10 ml DMEM supplemented with 10% FBS. Then, a mixture of 2.5 μg of the lentiviral ORF expression plasmid (i.e. Lenti-pEZ-Lv203) and 0.25 μg of the Lenti-Pac HIV plasmid in 15 μl of EndoFectin Lenti was incubated with 200 μl of Opti-MEM® (Invitrogen). The DNA-EndoFectin Lenti complex was directly added into the cultured cells before being allowed for overnight incubation at 37 °C in a CO_2_ incubator, and was then replaced by a fresh medium supplemented with 5% FBS. Subsequently, a 1/500 volume of the TiterBoost reagent was added to the culture medium and allowed to continue the cell culture, before the pseudovirus-containing culture medium 48 hours post transfection was collected by centrifuging at 500 × *g* for 10 min, and then the resulting lentivirus titer was estimated prior to being subjected to efficient transfection of target cells (i.e. *Nrf1α*^−*/*−^ hepatoma cells, being restored for stable expression of Nrf1α protein).

### The MTS assay

Experimental cells (5 × 10^3^) were allowed for growth for 24, 48, 72 and 96 h in each well of 96-well plates. The cells were washed with PBS and then incubated for additional 2 h with 100 μl of a mix of CellTiter96^®^ Aqueous One solution MTS [3-(4,5-dimethylthiazol-2-yl)-5-(3-carboxymethoxyphenyl)-2-(4-sulfophenyl)-2H-tetrazolium, from Promega] in the fresh DMEM containing 25 mM glucose and 10% FBS (V_MTS_:V_DMEM_ = 1:10), before the absorbance (λ = 490 nm) was measured by the Microplate Rader Model 680 (from Bio-Rad), in order to calculate cell viability that indirectly reflects the rate of cell proliferation. The results are graphically shown as the means ± S.D, which were calculated from at least three separate experiments that were each performed triplicate.

### Cell cycle and apoptosis analysis by flow cytometry

Experimental cells (5 × 10^5^) were allowed for growth in 60-mm cell culture plate for 48 h and synchronization by 12-h starvation in a serum-free medium, before being treated with 10 μmol/L BrdU for 12 h. The cell were fixed for 15 min with 100 μl of BD Cytofix/Cytoperm buffer (containing a mixture of the fixative paraformaldehyde and the detergent saponin) at room temperature and permeabilized for 10 min with 100 μl of BD Cytoperm permeabilization buffer plus (containing fetal bovine serum as a staining enhancer) on ice. Thereafter, the cells were re-fixed and treated with 100 μl of DNase (at a dose of 300 μg/ml in DPBS) for 1 h at 37 °C in order to expose the incorporated BrdU, followed by staining with FITC conjugated anti-BrdU antibody for 60 min at room temperature. Subsequently, the cells were suspended in 20 μl of 7-amino-actinomycin D solution 20 min for the DNA staining, and re-suspended in 0.5 ml of a staining buffer (i.e. 1 × DPBS containing 0.09% sodium azide and 3% heat-inactivated FBS), prior to the cell cycle analysis by flow cytometry. In addition, cells (5 × 10^5^) were allowed for 48-h growth in 60-mm cell culture plate before being harvested for apoptosis analysis. The cells were pelleted by centrifuging at 1000 × *g* for 5 min and washed by PBS three times, before being incubated for 15 min with 5 μl of Annexin V-FITC and 10 μl of propidium iodide (PI) in 195 μl of binding buffer, prior to apoptosis analysis by flow cytometry. The results shown herein were acquired using a low flow at 400 events per second, with the total events being 4 × 10^4^, before being analyzed by the FlowJo 7.6.1 software.

### Statistical analysis

The statistical significances of cell proliferation, migration, invasion and gene expression were determined using the Student’s *t* test or Two-way Analysis of Variations (ANOVA). The data are shown as a fold change (mean ± S.D or ± S.E), each of which represents at least 3 independent experiments that were each performed triplicate.

## Additional Information

**How to cite this article**: Ren, Y. *et al*. TALENs-directed knockout of the full-length transcription factor Nrf1α that represses malignant behaviour of human hepatocellular carcinoma (HepG2) cells. *Sci. Rep*. **6**, 23775; doi: 10.1038/srep23775 (2016).

## Supplementary Material

Supplementary Information

## Figures and Tables

**Figure 1:**
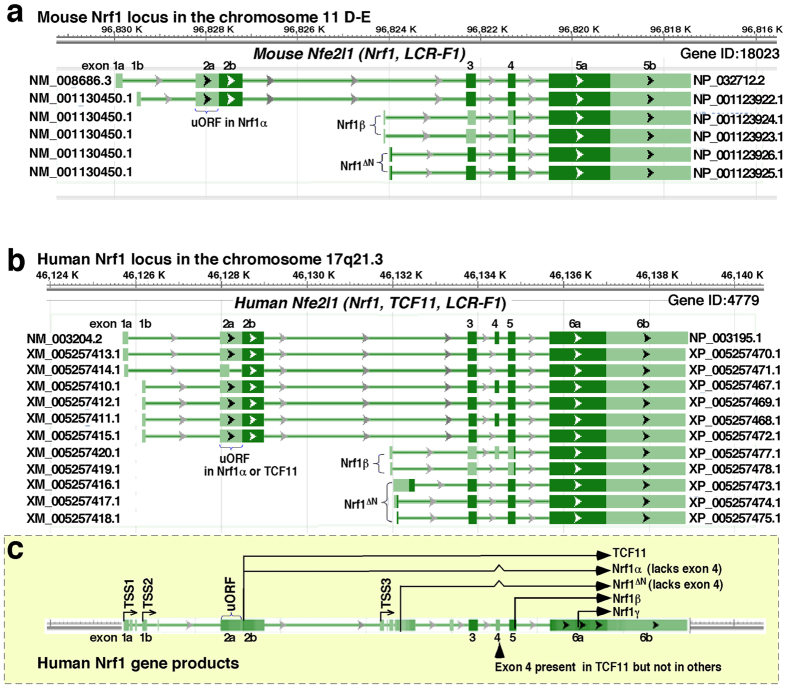
Schematics of the single *Nrf1* gene with its products of multiple transcript and polypeptide isoforms. Diagrammatic representation of chromosomal locations of the *Nfe2l1* gene loci (expressed as Nrf1, TCF11 and/or LCR-F1) in both the mouse (**a**) and human (**b**), with different numbers of their exons. The left-handed side shows different lengths of multiple transcripts with altered numbers of the exons indicated, which were predicted to translate various protein isoforms shown on the right-handed side. Of note, exon 2a is generally considered to be untranslated, but indeed is bioinformatically predicted to contain an upstream open reading frame (uORF), exons 3 to 5 located within the main ORF can also be allowed for no, partial or complete translation insomuch as to give rise to various lengths of distinct protein forms. (**c**) The schematic shows that production of multiple isofoms is predominantly attributable to alternative translation from mRNA variants arising from three different transcription start sites (e.g. to yield Nrf1α/TCF11, Nrf1^ΔN^ and Nrf1β), alternative splicing of longer transcripts (e.g. to remove exon 4 in Nrf1α and Nrf1^ΔN^), and the putative regulation of the long 3′-untranslational region (UTR) containing two polyA tail signals. The transcriptional expression is directed by arrows, whilst both untranslated and translated exons were represented by light and dark blue boxes, respectively. The site of the gene manipulated is specifically positioned in close proximity to the first translation start codons of Nrf1α.

**Table 1:** All pairs of F/R primers used for real-time qPCR.

Primer Name	Nucleotide sequences (5′ to 3′)
Actin-F	CATGTACGTTGCTATCCAGGC
Actin-R	CTCCTTAATGTCACGCACGAT
Nrf1-F	GCTGGACACCATCCTGAATC
Nrf1-R	CCTTCTGCTTCATCTGTCGC
CDH1-F	CGAGAGCTACACGTTCACGG
CDH1-R	GGGTGTCGAGGGAAAAATAGG
CDH2-F	TCAGGCGTCTGTAGAGGCTT
CDH2-R	ATGCACATCCTTCGATAAGACTG
VIM-F	GACGCCATCAACACCGAGTT
VIM-R	CTTTGTCGTTGGTTAGCTGGT
FN1-F	CGGTGGCTGTCAGTCAAAG
FN1-R	AAACCTCGGCTTCCTCCATAA
SNAI1-F	TCGGAAGCCTAACTACAGCGA
SNAI1-R	AGATGAGCATTGGCAGCGAG
SNAI2-F	CGAACTGGACACACATACAGTG
SNAI2-R	CTGAGGATCTCTGGTTGTGGT
CTNNA1-F	GGGGATAAAATTGCGAAGGAGA
CTNNA1-R	GTTGCCTCGCTTCACAGAAGA
CTNNB1-F	AAAGCGGCTGTTAGTCACTGG
CTNNB1-R	CGAGTCATTGCATACTGTCCAT
MMP9-F	TGTACCGCTATGGTTACACTCG
MMP9-R	GGCAGGGACAGTTGCTTCT
MMP17-F	CACTCATGTACTACGCCCTCA
MMP17-R	TGGAGAAGTCGATCTGGATGTC
CDK2-F	CCAGGAGTTACTTCTATGCCTGA
CDK2-R	TTCATCCAGGGGAGGTACAAC
CDK6-F	TCTTCATTCACACCGAGTAGTGC
CDK6-R	TGAGGTTAGAGCCATCTGGAAA
CCND1-F	GCTGCGAAGTGGAAACCATC
CCND1-R	CCTCCTTCTGCACACATTTGAA
P16-F	GATCCAGGTGGGTAGAAGGTC
P16-R	CCCCTGCAAACTTCGTCCT
P21-F	TGTCCGTCAGAACCCATGC
P21-R	AAAGTCGAAGTTCCATCGCTC
RB1-F	TTGGATCACAGCGATACAAACTT
RB1-R	AGCGCACGCCAATAAAGACAT
P53-F	CAGCACATGACGGAGGTTGT
P53-R	TCATCCAAATACTCCACACGC
CASP3-F	CATGGAAGCGAATCAATGGACT
CASP3-R	CTGTACCAGACCGAGATGTCA
CASP4-F	CAAGAGAAGCAACGTATGGCA
CASP4-R	AGGCAGATGGTCAAACTCTGTA
CASP6-F	ATGGCGAAGGCAATCACATTT
CASP6-R	GTGCTGGTTTCCCCGACAT
CASP9-F	CTCAGACCAGAGATTCGCAAAC
CASP9-R	GCATTTCCCCTCAAACTCTCAA
BCL2-F	GGTGGGGTCATGTGTGTGG
BCL2-R	CGGTTCAGGTACTCAGTCATCC
BCL2L1-F	GAGCTGGTGGTTGACTTTCTC
BCL2L1-R	TCCATCTCCGATTCAGTCCCT
