# Supplementary material for: TALENs-directed knockout of the full-length transcription factor Nrf1α that represses malignant behaviour of human hepatocellular carcinoma (HepG2) cells
Source: Sci Rep. 2016 Apr 11;6:23775. doi: 10.1038/srep23775 (PMC4827396; doi:10.1038/srep23775)

TALENs-directed knockout of the full-length transcription factor Nrf1α that represses malignant behaviour of human hepatocellular carcinoma (HepG2) cells

Yonggang Ren[1] Lu Qiu[1], Fenglin Lü[1], Xufang Ru[1], Shaojun Li[1], Yuancai Xiang[1], Siwang Yu[2], and Yiguo Zhang[1]*

[1]The Laboratory of Cell Biochemistry and Gene Regulation by Topobiology, College of Bioengineering and Faculty of Sciences, Chongqing University, No. 174 Shazheng Street, Shapingba District, Chongqing 400044, China;
[2]State Key Laboratory of Natural and Biomimetic Drugs, and Department of Chemical Biology, School of Pharmaceutical Sciences, Peking University Health Science Center, No 38 Xueyuan Rd., Haidian District, Beijing 100191, China;
*Correspondence should be addressed to **Yiguo Zhang** (**Email: yiguozhang@cqu.edu.cn**, **or eaglezhang64@gmail.com**)

SUPPLEMENTAL RESULTS

The main text of our present paper is accompanied by the supplemental data including six figures as shown herein. The first four figures (i.e. Figs. S1 to S4) reveal differences between three monoclonal cell lines of mono-allelic knockout (*Nrf1α*$^{+/-}$) and parent wild-type (*Nrf1α*$^{+/+}$) HepG2 cells. Of note, no further comparisons between the mono-allelic (*Nrf1α*$^{+/-}$) and bi-allelic (*Nrf1α*$^{-/-}$) knockout cell lines were presented here in the same experimental settings. The fifth figure shows changes in the basal constitutive expression of critical genes that are required for controlling cancer cell process (e.g. EMT) and malignant behaviour (e.g. invasion and metastasis), as well as those involved in the regulation of the cell division cycle and apoptosis (see Fig. S5), in the subcutaneous tumour xenograft mice that had been injected with either *Nrf1α*$^{-/-}$ or *Nrf1α*$^{+/+}$ hepatoma cells. Lastly, Fig. S6 illustrates histopathological staining of the human hepatocellular carcinoma tissues.

Figure S1

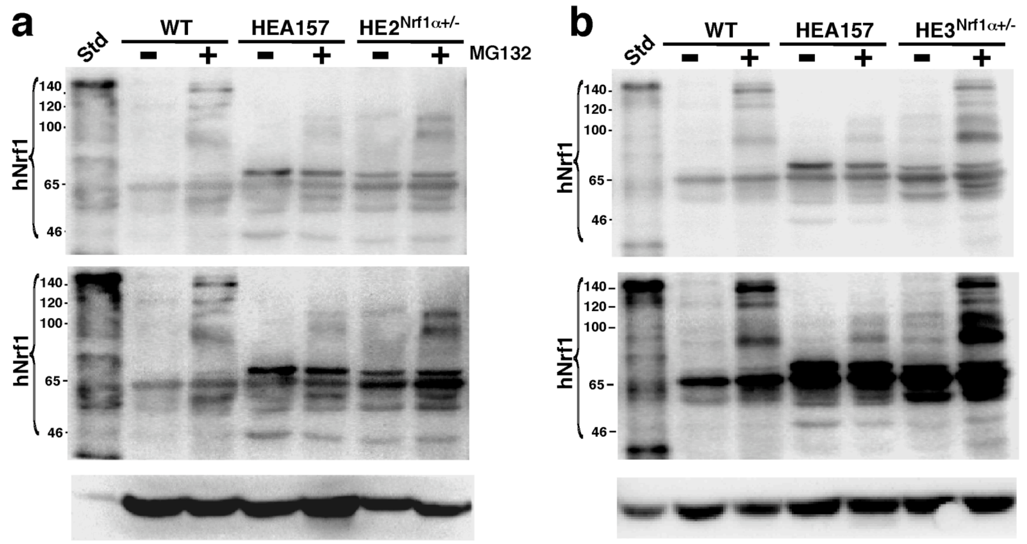

Figure S1. Western blotting to identify additional two monoclonal cell lines of mono-allelic knockout (*Nrf1α*$^{+/-}$).
Three mono-allelic knockout (*Nrf1α*$^{+/-}$) monoclonal cell lines (called HE1$^{Nrf1α+/-}$, HE2$^{Nrf1α+/-}$ and HE3$^{Nrf1α+/-}$) had been selected by a series of cell-subcloning cultures and identified by target cDNA sequencing, western blotting and other technologies. The results of western blotting with anti-Nrf1 antibodies have been presented herein (**a**,**b**) and in the main text (see Fig. 4**b**,**c**), which are a representative of at least three independent experiments undertaken on separate occasions that were each performed in triplicate. The upper two images were obtained from different periods of time while the Nrf1-immunoblotted membranes were exposed to X-ray. WT, indicates intact wild-type (*Nrf1α*$^{+/+}$) HepG2 cell lysates, whilst Std represents ectopically Nrf1-expressing HepG2 cell lysates, in addition to the bi-allelic knockout (*Nrf1α*$^{-/-}$) HEA157 cells that served as a positive control.

Figure S2

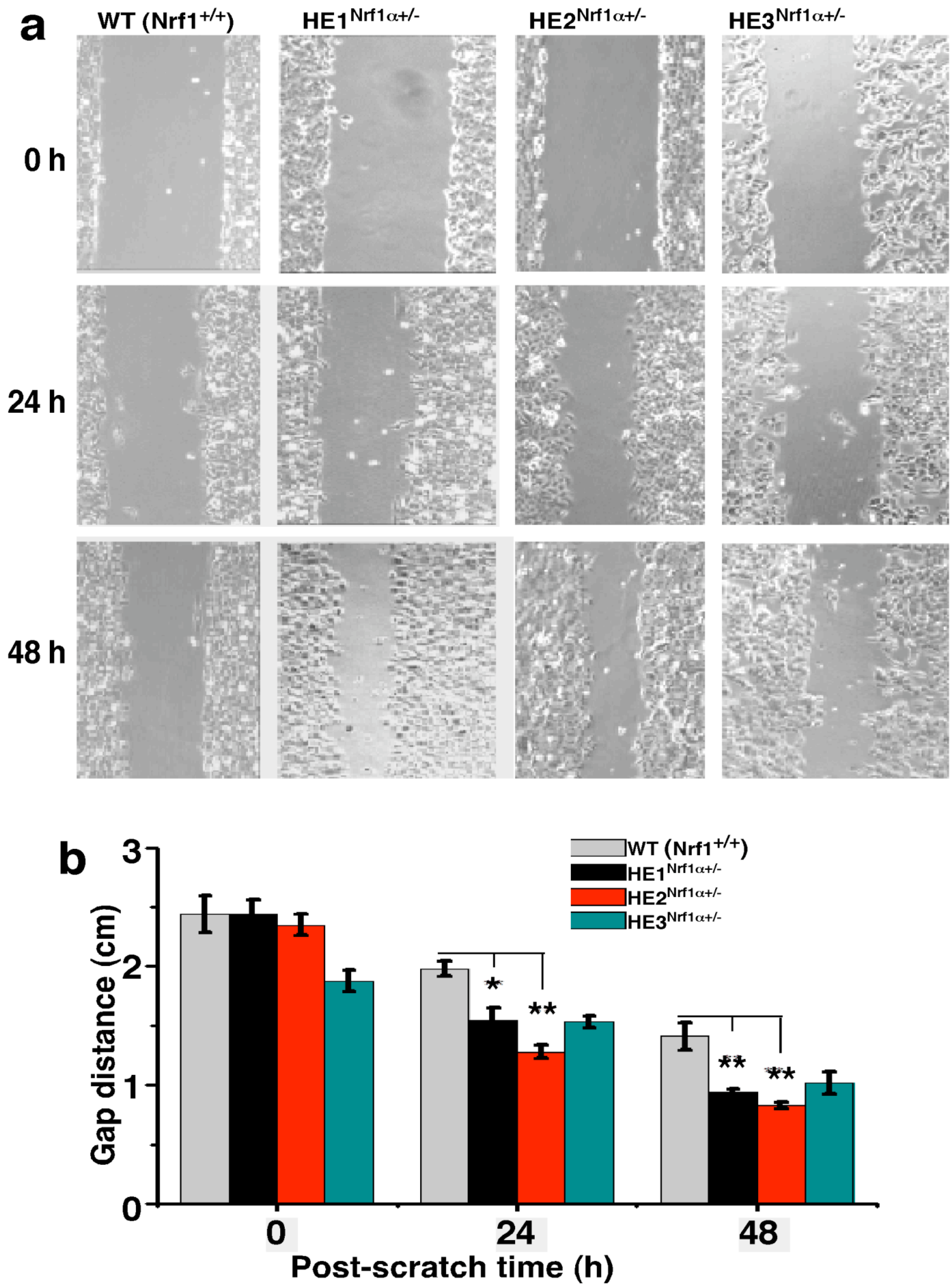

Figure S2. Differences in migration of between three *Nrf1*α$^{+/-}$ cell lines to close the *in vitro* scratch.

(**a**) Three heterozygous knockout monoclonal cell lines (HE1$^{Nrf1\alpha+/-}$, HE2$^{Nrf1\alpha+/-}$ and HE3$^{Nrf1\alpha+/-}$), together with wild-type (*Nrf1α*$^{+/+}$) HepG2 cells, were starved for 12 h in a serum-free medium and then treated for additional 6 h with 1μg/ml of mitomycin C. Subsequently, a clear "scratch" was created before being allowed for being healed in the continuous culture at 37℃ with 5% CO2. The scratched images were captured at the beginning and at 12-h intervals during cell migration to close the scratch, followed by quantification of the distance of cell migration. (**b**) The results were calculated as a fold change (mean ± S.D.) of the scratched gap distance of *Nrf1α*$^{+/-}$ cells, which are shown as a representative of three independent experiments undertaken on separate occasions that were each performed in triplicate. Significant decreases (*$p<0.05$, **$p<0.01$, n=9) are indicated, relative to the corresponding control values measured from wild-type (*Nrf1α*$^{+/+}$) HepG2 cells.

Figure S3

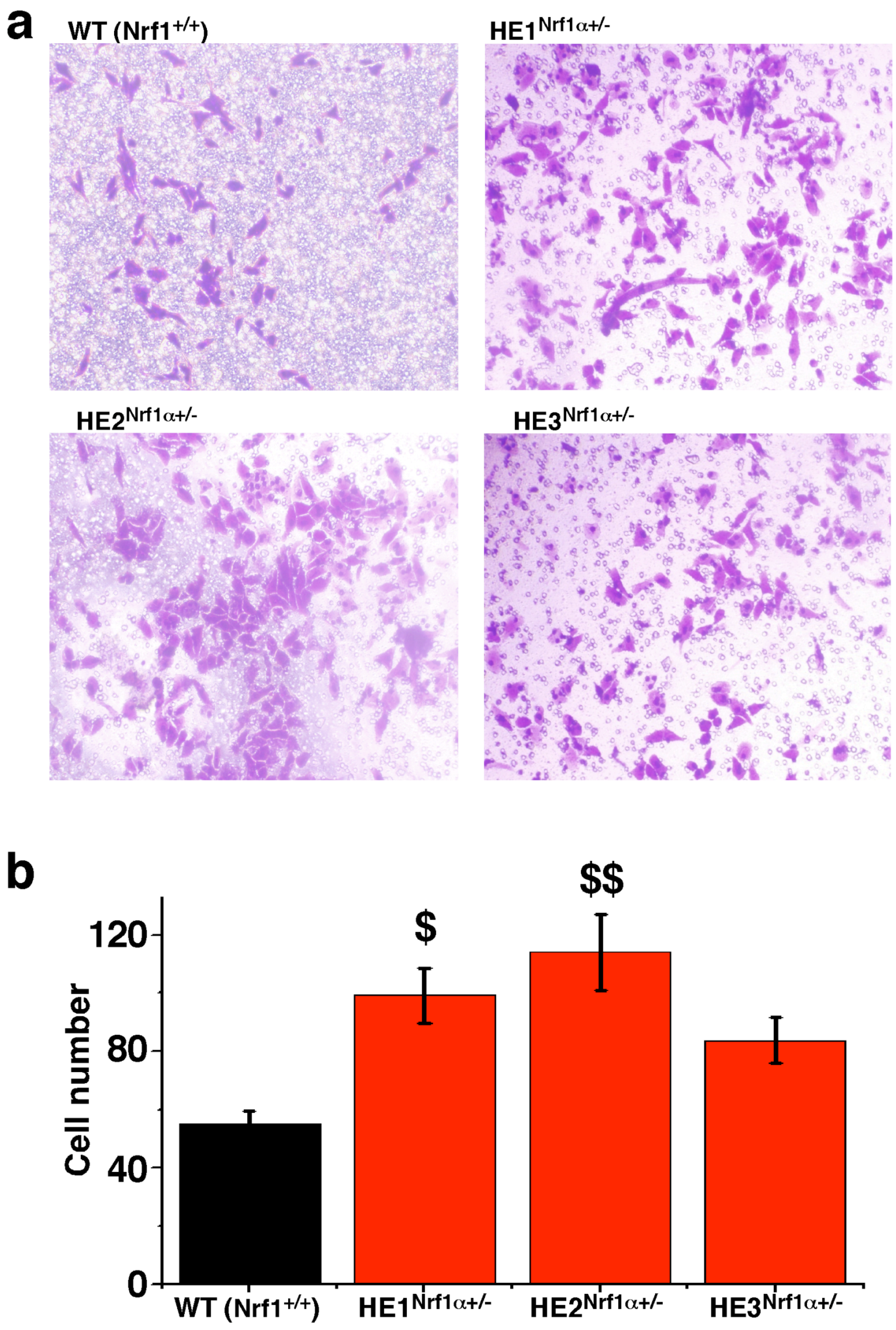

Figure S3. Distinct *Nrf1*$\alpha^{+/-}$ cell lines with different migratory potentials to pass through transwells.

(**a**) Three mono-allelic knockout cell lines (HE1$^{Nrf1\alpha+/-}$, HE2$^{Nrf1\alpha+/-}$ and HE3$^{Nrf1\alpha+/-}$), along with wild-type (*Nrf1*$\alpha^{+/+}$) HepG2 cells were starved for 12 h in a serum-free medium and then subjected to transwell migration assays as described in the section of 'Materials and methods' (see the main text). The migratory cells, that had passed through the 8-μm microporous membrane and attached to the lower surface of the transwell membranes, were fixed with 4% paraformaldehyde and stained with 1% crystal violet reagent before being counted. (**b**) The results were calculated as a fold change (mean ± S.D.) of migratory *Nrf1*$\alpha^{+/-}$ cells, which are shown as a representative of at least three independent experiments undertaken on separate occasions that were each performed in triplicate. Significant increases ($^{\$}p<0.05$, n=9) are indicated, relative to the corresponding control values obtained from wild-type *Nrf1*$\alpha^{+/+}$ HepG2 cells.

Figure S4

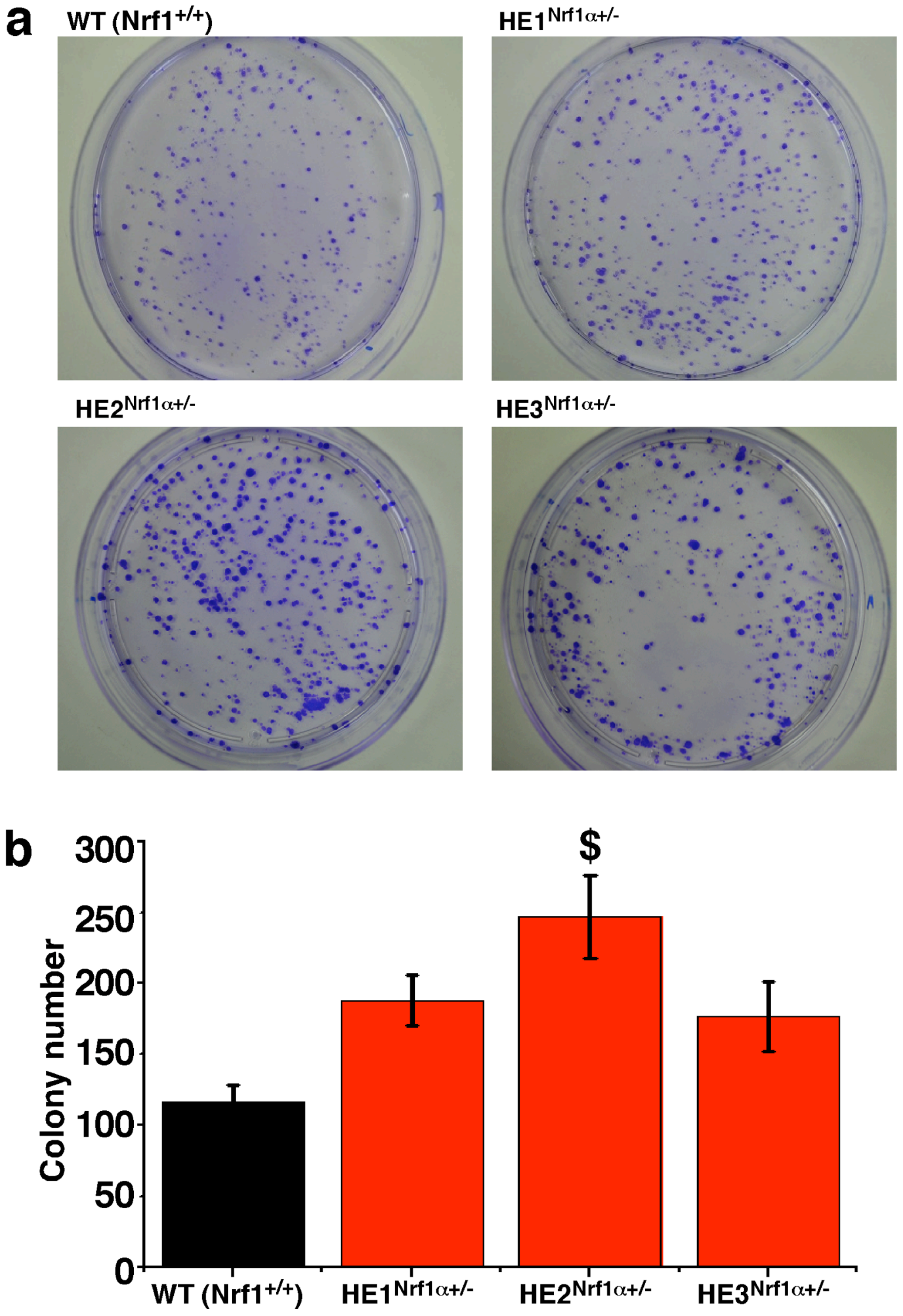

Figure S4. Differences in their clone formation of distinct *Nrf1*α$^{+/-}$ cell lines on soft agar gels.

(**a**) The soft agar colony formation of the above mono-allelic knockout cell lines (HE1$^{Nrf1\alpha+/-}$, HE2$^{Nrf1\alpha+/-}$ and HE3$^{Nrf1\alpha+/-}$), along with wild-type (*Nrf1α*$^{+/+}$) HepG2 cells, was examined as described in the text of 'Materials and methods'. The resulting cell clones formed on the soft agar plates were stained with 1% crystal violet reagent before being counted. (**b**) The data were calculated as a fold change (mean ± S.D.) of the number of *Nrf1α*$^{+/-}$cell clone formation, and the significant increase ($^{\$}p<0.05$, n=9) is analyzed, relative to the control values of *Nrf1α*$^{+/+}$ cells. There are shown herein as a representative of at least three independent experiments undertaken on separate occasions that were each performed in triplicate.

Figure S5

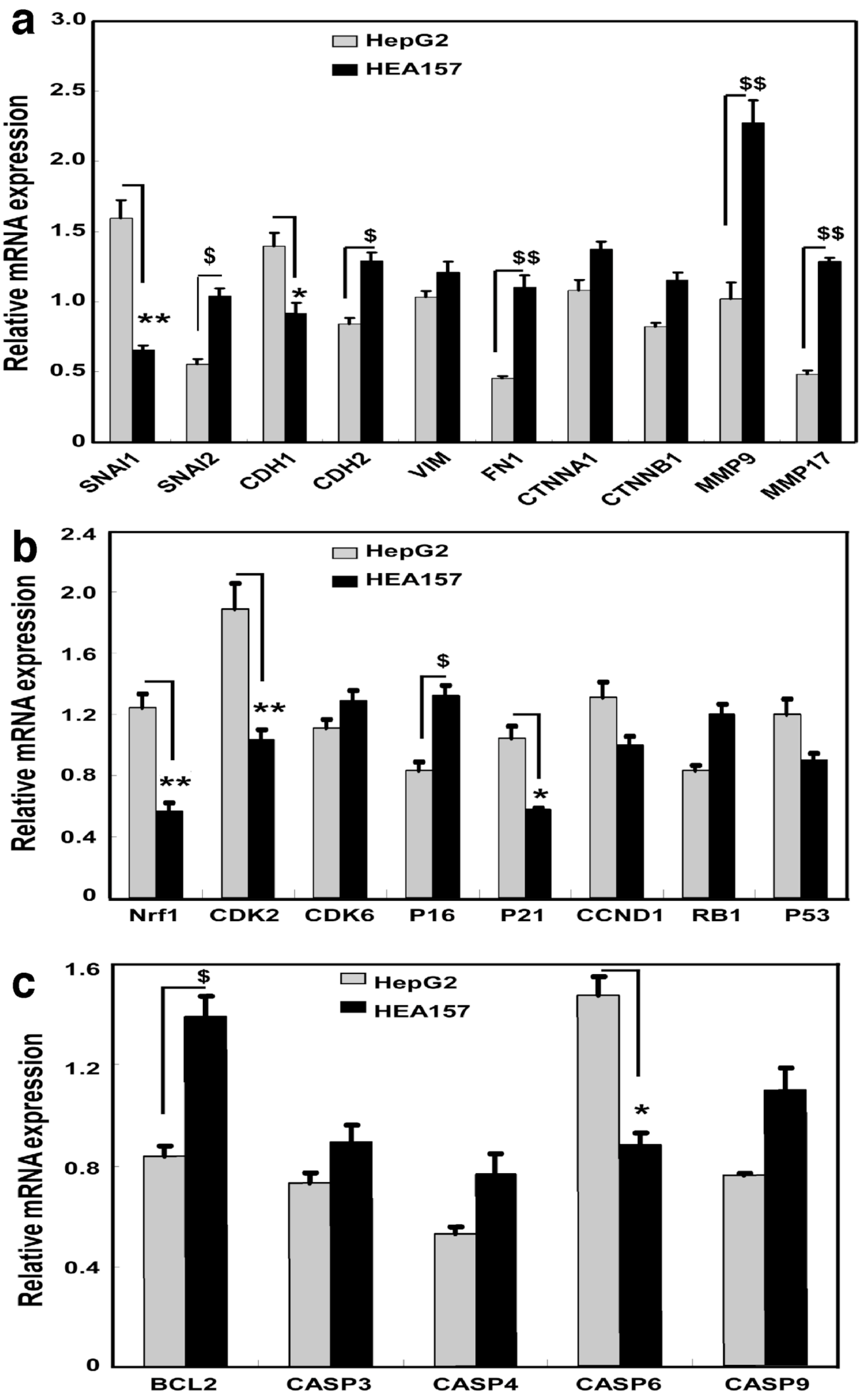

Figure S5. Real-time qPCR analysis of critical genes dysregulated in *Nrf1*$\alpha^{-/-}$ cells-derived xenograft tissues.

Either HepG2 ($Nrf1\alpha^{+/+}$) or HEA157 ($Nrf1\alpha^{-/-}$) cells that that had been growing in the exponential phase were inoculated subcutaneously into male nude mice, followed by observation of the subcutaneous tumour xenografts that had emerged and developed (see Fig. 8 in the main text). Until six weeks when the mice were sacrificed, the xenograft tumors were excised and then subjected to both histopathological examinations (see Fig. 10 in the main text) and real-time qPCR analysis (herein). Total RNAs were isolated from the xenograft tumours derived from HepG2 ($Nrf1\alpha^{+/+}$) or HEA157 ($Nrf1\alpha^{-/-}$) hepatoma cells and then reversely transcribed into the first strand of cDNA. Subsequently, different mRNA expression levels of distinct genes controlling cancer cell process and malignant behaviour [i.e. migration and invasion including the EMT markers (**a**), the cell division cycle (**b**), and apoptosis (**c**),] were measured by quantitative real-time PCR. The results were calculated as a fold change (mean ± S.E) of mRNA levels of gene expression in $Nrf1\alpha^{-/-}$ cells and revealed that loss of Nrf1α results in dysregulation of indicated gene transcription with significant decreases (*p<0.05, n=9) or significant increases ($^{\$}$p<0.05, $^{\$\$}$p < 0.01, n=9), relative to their basal mRNA levels of corresponding genes expressed in wild-type $Nrf1\alpha^{+/+}$ cells. These data shown here are a representative of at least three independent experiments undertaken on separate occasions that were each performed in triplicate.

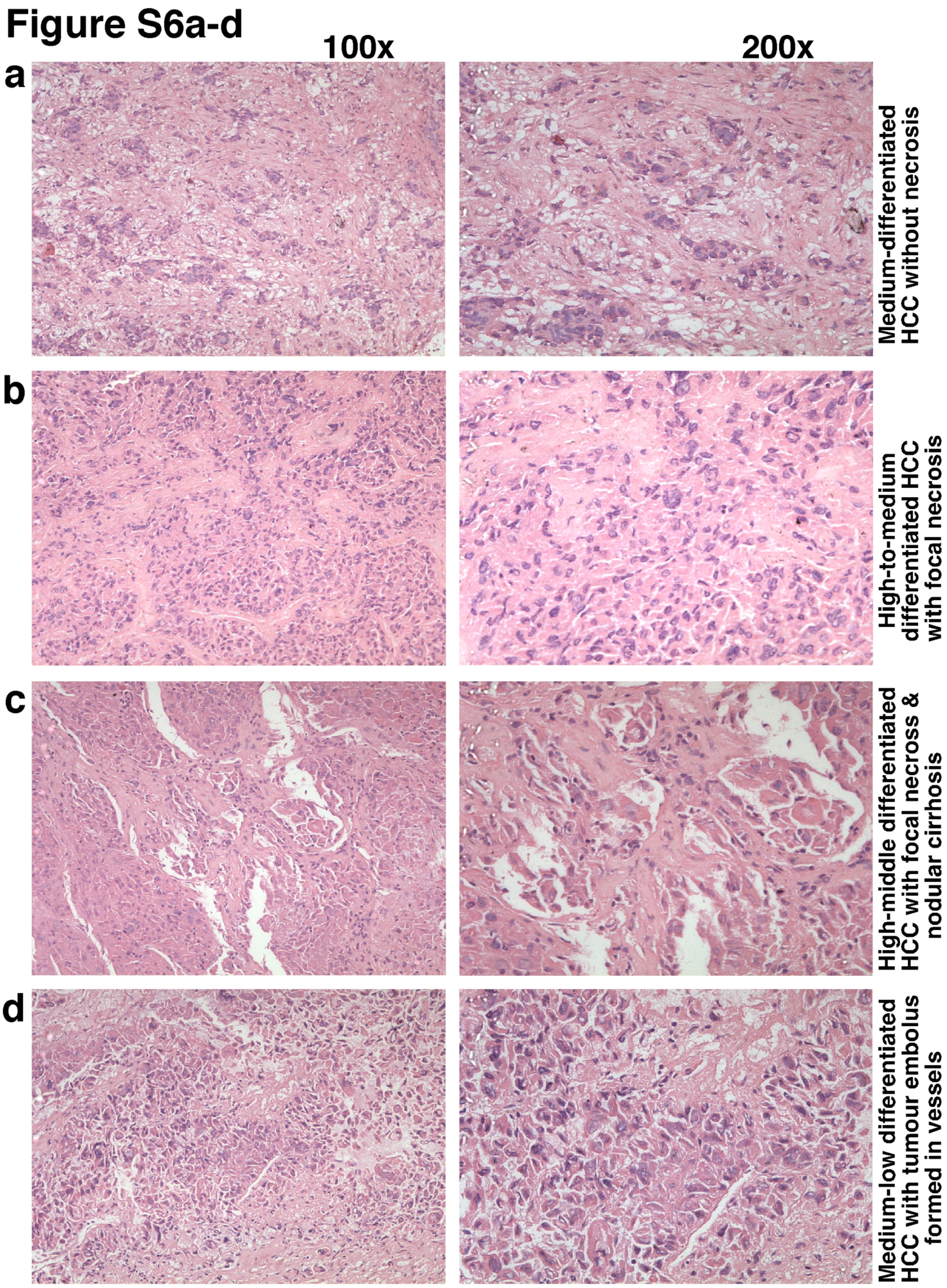

Figure S6. Histopathological examinations of the human hepatocellular carcinoma concomitantly with chronic lesions. All seven human hepatocellular carcinoma (HCC) are surgically sampled from the Third Hospital Affiliated to the Third Military Medical University, Chongqing, China. The Informed Consent was given for the permission by each of the patients before all samples were histopathologically confirmed prior to RT-qPCR, western blotting and immunohistochemistry (see Fig. 14 in the main text). All relevant protocols used in this study had been approved by the hospital's Protection of Human Subjects Committee. The above seven samples of HCC were re-examined by the routine hematoxylin-eosin staining (HE) and ensuing histopathological analysis. For detailed descriptions, please see the relevant main text.

Figure S6efg

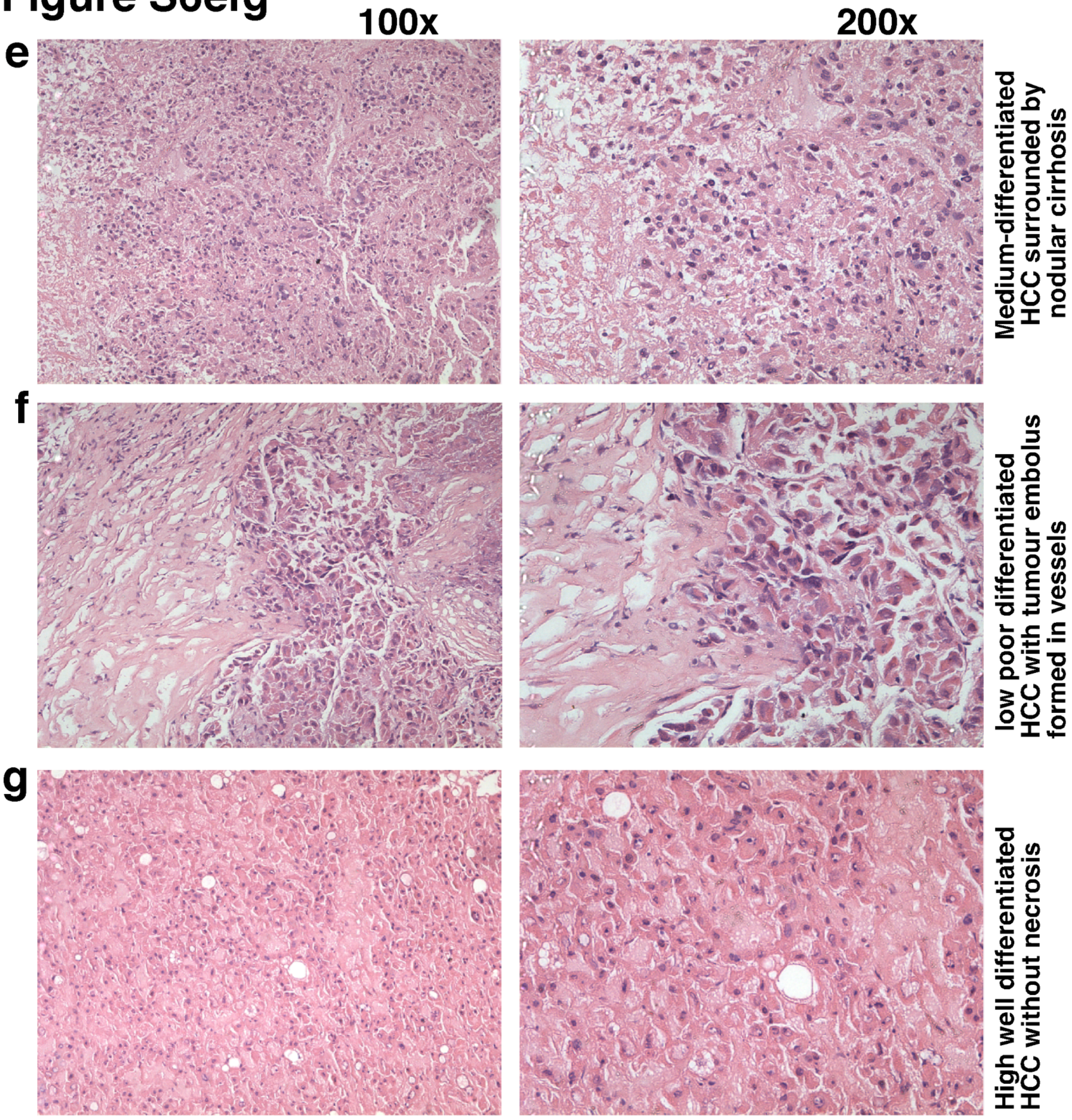

Supplement: Supplementary Information [file srep23775-s1.pdf]
